# Does gender expression matter in tailoring anti-smoking messages for sexual and gender minority young adults?

**DOI:** 10.1093/ntr/ntag026

**Published:** 2026-02-13

**Authors:** Qijia Ye, Jiaxi Wu, Elaine Hanby, N F N Scout, Bob Gordon, Julia Applegate, Ana Machado, Sixiao Liu, Juno Obedin-Maliver, Mitchell R Lunn, Jennifer Potter, Jarvis T Chen, Shoba Ramanadhan, Kasisomayajula Viswanath, Andy S L Tan

**Affiliations:** Annenberg School for Communication, University of Pennsylvania, Philadelphia, PA, USA; Annenberg School for Communication, University of Pennsylvania, Philadelphia, PA, USA; Annenberg School for Communication, University of Pennsylvania, Philadelphia, PA, USA; National LGBT Cancer Network, Providence, RI, USA; California LGBT Tobacco Education Partnership, San Francisco, CA, USA; Department of Women’s, Gender and Sexuality Studies, The Ohio State University, Columbus, OH, USA; CenterLink Inc, The Community of LGBTQ Centers, Fort Lauderdale, FL, USA; Department of Population Health Sciences, College of Medicine, University of Central Florida, Orlando, FL, USA; Department of Obstetrics and Gynecology, Stanford University School of Medicine, Stanford, CA, USA; The PRIDE Study/PRIDEnet, Stanford University School of Medicine, Stanford, CA, USA; Department of Epidemiology and Population Health, Stanford University School of Medicine, Stanford, CA, USA; The PRIDE Study/PRIDEnet, Stanford University School of Medicine, Stanford, CA, USA; Department of Epidemiology and Population Health, Stanford University School of Medicine, Stanford, CA, USA; Division of Nephrology, Department of Medicine, Stanford University School of Medicine, Stanford, CA, USA; The Fenway Institute, Boston, MA, USA; Department of Medicine, Harvard Medical School, Boston, MA, USA; Division of General Internal Medicine, Beth Israel Lahey Health, Boston, MA, USA; Department of Social and Behavioral Sciences, Harvard T.H. Chan School of Public Health, Boston, MA, USA; Department of Social and Behavioral Sciences, Harvard T.H. Chan School of Public Health, Boston, MA, USA; Department of Social and Behavioral Sciences, Harvard T.H. Chan School of Public Health, Boston, MA, USA; Divison of Population Sciences, Dana-Farber/Harvard Cancer Center, Boston, MA, USA; Annenberg School for Communication, University of Pennsylvania, Philadelphia, PA, USA; Leonard Davis Institute of Health Economics, University of Pennsylvania, Philadelphia, PA, USA; Abramson Cancer Center, Tobacco and Environmental Carcinogenesis Program, Philadelphia, PA, USA

## Abstract

**Introduction:**

Smoking rates among sexual and gender minority (SGM) adults are generally higher than those in the general population. Tailoring anti-smoking messages for SGM young adults is a promising strategy to reduce health inequalities, but ignoring the difference between gender groups within the SGM community is problematic.

**Methods:**

We conducted two experiments to examine whether gender expression affected the perceived targetedness (PT) among SGM young adults aged 18 to 30. In the first study, participants were randomly assigned to view 8 out of 80 images using various gender expressions. In the second study, participants were randomly assigned to one of four conditions (transgender/nonbinary expressions vs. feminine expressions vs. masculine expressions vs. multiple gender expressions) and viewed six anti-smoking campaign messages in each condition.

**Results:**

Both studies found that compared to messages with other gender expressions, gender-expansive individuals reported higher PT when viewing messages with transgender/nonbinary expressions. Cisgender sexual minority women reported higher PT when viewing messages with feminine expressions. Cisgender sexual minority men reported higher PT when viewing messages with masculine expressions. There was no significant difference in PT between multiple gender expressions and gender expressions matching individuals’ gender identity.

**Conclusions:**

Culturally tailored anti-smoking messages using gender expressions matched to gender identity or messages using multiple gender expressions were more effective in achieving PT than messages using unmatched gender expressions.

**Implications:**

Gender is critical to tailoring anti-smoking campaign messages for SGM young adults. We should not view the SGM community as a monolith in smoking interventions and need to tailor messages to different gender groups within the community. Future research should investigate how sexual expressions affect PT and how gender expressions influence persuasion outcomes beyond PT. Additionally, intersectional analyses of multiple social identities may provide deeper insights into the effects of SGM-tailored anti-smoking messages.

## Introduction

Sexual and gender minority (SGM) adults report higher rates of cigarette smoking than the general adult population.^1^^,^^2^ In particular, young gay/lesbian and bisexual women (vs. heterosexual women), young gay men (vs. heterosexual men),^3-5^ and transgender boys (vs. cisgender boys) smoke cigarettes at higher rates.^6^ Multiple factors lead to these disparities in smoking between SGM young adults and their peers. The tobacco industry has targeted and marketed to the SGM community for decades.^7^ SGM individuals who smoke report higher rates of tobacco advertising exposure than non-SGM individuals.^8^ Moreover, minority stressors such as stigma and internalized homophobia increase the risk of smoking^9^ and smoking-related diseases such as stroke and lung cancer^10^ among SGM young adults. The health inequalities call for effective smoking interventions for SGM young adults.

### Culturally tailored anti-smoking messages

A promising strategy to reduce health inequalities is tailoring anti-smoking messages for SGM young adults. Culturally tailored messages match the needs and preferences of a social group’s characteristics, such as identities, values, and norms^11^^,^^12^ to resonate with members in the group^13^ (e.g., using SGM-related imagery, symbols, and language as peripheral cues tailored for SGM). Message receivers often perceive the tailored messages as personally relevant and acceptable because of the congruency between characteristics of people and messages.^12^^,^^14^ Therefore, culturally tailored messages may be more effective in changing health behaviors than non-tailored messages.^15^

Although SGM individuals prefer culturally tailored messages to general health messages,^16^^,^^17^ the effectiveness of SGM-tailored anti-smoking messages is unclear.^18^ SGM-tailored group-based smoking interventions can improve short-term smoking cessation.^19^ This *Free Life*, the first large-scale SGM-tailored anti-smoking campaign in the United States, was effective in changing some smoking-related beliefs.^20^^,^^21^ However, compared to non-tailored messages, SGM-tailored anti-smoking messages did not significantly lower intention to purchase cigarettes or increase intention to quit smoking among sexual minority women (SMW) who currently smoked.^22^ Perceived message effectiveness of SGM-tailored counter-industry tobacco messages were not different from non-tailored messages.^23^ The effectiveness of SGM-tailored messages may depend on multiple contextual factors, such as how the message is tailored for an audience.

### Issues in tailoring anti-smoking messages

Culturally tailored anti-smoking messages often address the SGM community as a monolithic audience, which is an ineffective segmentation strategy. First, there are differences in smoking rates within the SGM community. Cisgender SMW, including lesbian and bisexual women, are more likely to use any tobacco product than cisgender gay men.^24^ Bisexual women (predominantly cisgender) smoke at higher rates than lesbian women.^25^ Furthermore, although the visibility of SGM individuals has increased over the years in mainstream media, lesbian women, bisexual people, and gender minorities (e.g., gender-expansive and transgender individuals) are historically less portrayed than gay men.^26^ Similarly, anti-smoking interventions were more likely to be tailored for gay men,^27^ creating a gap in tailored interventions for other SGM subgroups.

According to the distinctiveness theory,^28^ individuals who belong to a distinctive social group (eg, SGM group) are sensitive to the characteristics (i.e., gender and sexuality) shared by that group spontaneously because those characteristics are more central to the self-concept than other characteristics. Messages presenting those distinctive characteristics could be highly tailored and effective. Because gender and sexuality are distinctive characteristics that define SGM subgroups, our study examines whether SGM young adults tend to perceive being targeted by culturally tailored messages based on gender and sexuality expressions. It is essential to evaluate how these expressions in SGM-tailored anti-smoking messages affect perceived targetedness (PT), message receivers’ belief that they are the intended audience for a message.^29^ However, few studies have examined PT in tailoring anti-smoking campaign messages for SGM young adults.^23^

### The role of gender in tailoring anti-smoking messages

As formative research for Project RESIST,^30^^,^^31^ this study examines how visually distinct gender expressions influence PT in tailoring anti-smoking campaign messages for SGM young adults. Gender expression is defined as the external presentation of gender identity, and gender identity refers to an individual’s sense of their gender.^32^ We contend that a match between message gender expression and individual gender identity will optimize the perception that message receivers are the targeted audience. Gender identity is a central characteristic of many SGM individuals’ experiences, and gender expression is a salient message feature that elicits a comparison between the message receiver’s characteristics and message characteristics. Specifically, we hypothesize that gender-expansive individuals (ie, individuals who identify at least one nonconforming/nonbinary gender identity) will report higher PT when viewing messages using transgender/nonbinary expressions than messages using other gender expressions (**H1**); Cisgender SMW will report higher PT when viewing messages using feminine expressions than messages using other gender expressions (**H2**); Cisgender sexual minority men (SMM) will report higher PT when viewing messages using masculine expressions than messages using other gender expressions (**H3**).

The effects of gender expressions in health messages on PT among transgender individuals remain unknown. Trans feminine individuals (i.e., individuals who identify as transgender women) have different gender identities and life experiences than trans masculine individuals (i.e., individuals who identify as transgender man).^33^^,^^34^ Other than transgender/nonbinary expressions, trans feminine individuals may also perceive high similarity with feminine expressions in health messages. In contrast, trans masculine individuals may perceive high similarity with masculine expressions in health messages. This assertion lacks empirical evidence. Therefore, we posed two research questions: Will trans feminine individuals report higher PT when viewing messages using transgender/nonbinary expressions and feminine expressions compared with messages using other gender expressions? (**RQ1**) Will trans masculine individuals report higher PT when viewing messages using transgender/nonbinary expressions and masculine expressions compared with messages using other gender expressions? (**RQ2**).

Additionally, we explore how SGM individuals respond to messages depicting a group of people who represent different gender expressions. Although messages using multiple gender expressions would be more inclusive of various groups within the SGM community, we do not know the extent to which SGM individuals perceive being targeted by this type of message compared with those that match their gender identity. We therefore pose a third question: Will SGM individuals rate the PT of messages using multiple gender expressions similarly to the PT of messages using gender expressions matching their gender identity? (**RQ3**).

Two experiments were conducted to test the hypotheses and answer the research questions. In study 1, we adopted a mixed factorial experimental design using images representing different gender expressions. To improve research validity for future anti-smoking intervention design and replicate the findings of study 1, we conducted study 2, which adopted a between-subject experimental design using images from study 1 paired with anti-smoking arguments as message stimuli.

## Materials and methods

### Study 1: Image study

#### Participant recruitment

We recruited participants from the Prolific, a survey research platform that provides access to large and diverse populations and conducts stringent verification of survey respondents ensure data quality. Eligible participants were U.S. SGM young adults aged 18 to 30 years old. We recruited a convenience sample of 461 eligible participants, stratified by gender group so that approximately one-third of them were gender-expansive/transgender individuals, cisgender SMW, and cisgender SMM, respectively.

#### Study procedure and data collection

We used a mixed factorial experimental design. We invited eligible participants to complete a survey using the Qualtrics platform. After consent, participants rated eight images randomly selected from a pool of 80 images categorized into four gender expressions: transgender/nonbinary, feminine, masculine, or multiple gender expressions. Participants rated PT and other variables (e.g., image liking) of each image, which they were briefed that those images would be used in anti-tobacco messaging. Data were collected in January 2023.

#### Images and gender expressions

All the images used in the study comprised original photos and stock photos presenting young adults with diverse gender expressions. Characters in the original photos were volunteers who self-identified as SGM. The volunteers were not involved in the study as research participants. Stock photos were obtained from an online image database, where each photo was metadata-labeled with a gender identity, including “transgender/nonbinary,” “women,” “men,” and “mix.” To preserve authenticity of individuals’ self-identification, we categorized the images into four gender expressions based on self-reported identities or metadata labels, including transgender/nonbinary expressions, feminine expressions, masculine expressions, and multiple gender expressions. Characters in images generally expressed a positive state (e.g., smiling face). The images varied in multiple features (e.g., type of portrait and image orientation). See *Study 1 Supplementary Materials*  Figure S1 for examples of images. Table S2 shows that compared with images using transgender expressions, participants were less likely to perceive the characters as transgender when viewing images with masculine or feminine expressions, suggesting that the manipulation of gender expressions was partially effective.

#### Measures

We assessed gender identity with options adapted from the Population Research in Identity and Disparities for Equality (PRIDE) Study^35^ and grouped participants into five mutually exclusive gender categories for analyses: (a) Gender-expansive individuals, (b) Trans feminine individuals, (c) Trans masculine individuals, (d) Cisgender SMW, and (e) Cisgender SMM.^36^ We also measured participants’ perception of LGBT identification of content in the image, sexual orientation, and other demographics (eg, age and race). We assessed PT on a three-item 5-point Likert scale^29^ (Cronbach’s *a* = .97, *M* = 3.14, *SD* = 1.22). See Table 1 for specific measures.

**Table 1:** Measures

Constructs	Items
Gender identity^a^	How do you currently identify? Please select all that apply. 1 = Agender 2 = Cisgender Man 3 = Cisgender Woman 4 = Genderqueer 5 = Man 6 = Nonbinary 7 = Questioning 8 = Transgender Man 9 = Transgender Woman 10 = Woman 11 = Another gender identity (please specify)
Sex assigned at birth	What sex were you assigned at birth, on you original birth certificate? 1 = Male 2 = Female 3 = Intersex
LGBT image perception^b^	Did the following come to mind when you saw this health message? Please choose all that apply. 1 = White people 2 = Black people 3 = People of Hispanic or Latinx origin 4 = Asian people 5 = LGBT individuals 6 = Gays or lesbians 7 = Bisexuals 8 = Transgender and gender expansive people 9 = Straight people 10 = Upper class people 11 = Middle class people 12 = Working class people 13 = Poor people 14 = None of the above
Sexual orientation^c^	Do you consider yourself to be:(Choose all that apply) 1 = Asexual 2 = Bisexual 3 = Gay 4 = Lesbian 5 = Pansexual 6 = Queer 7 = Questioning 8 = Same-gender loving 9 = Straight/heterosexual 10 = Another sexual orientation (please specify)
Perceived targetedness	1. I would feel this message was intended for people like me. 2. I would believe I was in the target audience the designer created the health message for. 3. I would think the designer made the health message to appeal to people like me. 1 = Strongly disagree 2 = Disagree 3 = Neither agree nor disagree 4 = Agree 5 = Strongly agree

a The gender-expansive group included participants who identified at least one nonconforming/nonbinary gender (i.e., agender, genderqueer, nonbinary, questioning, and two-spirit) and provided a written description of a nonconforming/nonbinary gender identity regardless of sex assigned at birth. The trans feminine group included participants who identified as transgender woman and those who identified as woman and were assigned male at birth. The trans masculine group included participants who identified as transgender man and those who identified as man and were assigned female at birth. Cisgender SMW included participants who identified as a sexual minority person, cisgender woman, and woman and were assigned female at birth. Cisgender SMM included participants who identified as a sexual minority person, cisgender man, and man and were assigned male at birth.

b When participants selected “LGBT individuals,” “Gays or Lesbians,” “Bisexuals,” or “Transgender and gender-expansive people,” we coded LGBT image perception as “Yes.” Otherwise, they were coded as “No.”

c We grouped participants into three sexual orientation categories: “Gay or lesbian,” “Bisexual,” and “Other.” “Gay or lesbian” included participants who only selected “Gay” or “Lesbian.” “Bisexual” included participants only selected “Bisexual.” “Other” included participants who selected the rest of or multiple sexual orientations.

#### Data analysis

We used cross-classified multilevel modeling to examine the effects of gender expression and gender identity on PT because observations were structured within images and individuals. A multilevel model predicted PT using image gender expression and respondent gender identity as predictors, image characteristics (LGBT image perception and number of characters in image), and individual characteristics (age, sexual orientation, race, education, income, marital status, and smoker status) as covariates, and random effects at the image level and the individual level. We examined the matching effect of image gender expression and respondent gender identity by adding their interaction term to the main effect model. We estimated marginal means of PT, setting continuous covariates to their respective means and categorical covariates’ responses to their respective proportions. Analyses were conducted in R using the “lme4” package.^37^

### Study 2: Anti-smoking message study

#### Participant recruitment

Eligible participants were U.S. SGM young adults aged 18 to 30 years old. We recruited a convenience sample of 1247 U.S. SGM young adults aged 18 to 30 from the Prolific 2 months after study 1. Participants were stratified by gender group so that approximately one-third of them were gender-expansive/transgender individuals, cisgender SMW, and cisgender SMM, respectively. After excluding 57 participants who failed attention checks or did not complete most survey items, we obtained a final sample of 1190 participants for this analysis.

#### Study procedure and data collection

The study used a between-subject experimental design. After providing informed consent, eligible participants were randomly assigned to one of four experimental groups (transgender/nonbinary expressions vs. feminine expressions vs. masculine expressions vs. multiple gender expressions) using the Qualtrics built-in randomizer function. Each participant viewed six anti-smoking campaign messages within their assigned group of messages. The messages were presented in random order. Participants were asked to rate the PT of each message. Data were collected between March 2023 and May 2023.

#### Anti-smoking messages

The anti-smoking messages consist of two main components: an image picturing SGM individuals and a text presenting an anti-smoking argument. Experimental groups only differed in gender expression represented by images. We selected the six top-ranked images in each gender expression category from study 1 based on combined evaluations across four criteria: image liking, character similarity, cultural sensitivity, and minimal anger induction. The six arguments were used in each experimental condition, and each was used once. All messages used an identical message template that consisted of a QR code accessing information of quitting smoking, a logo, and a slogan of our project (“LGBTQ+ health is our focus.”). The messages were reviewed by an advisory committee comprising leaders of LGBT non-profit organizations and experts of SGM health to ensure they were culturally tailored to the SGM community and unlikely to elicit strong psychological reactance. *Study 2 Supplementary Materials*  Figure S2 shows all stimuli in each condition. Table S6 shows that transgender/nonbinary expressions elicited stronger transgender image perception than the other three gender expressions, suggesting that the manipulation of gender expressions was partially effective.

#### Measures

The measures of gender identity, LGBT image perception, sexual orientation, and PT (Cronbach’s *a* = .95, *M* = 3.15, *SD* = .95) were identical to those used in study 1.

#### Data analysis

We computed the average score of PT across within-group messages for each participant. LGBT image perception was calculated as the proportion of messages identified as including an LGBT character in the condition. Multiple linear regression was performed to examine the effects of message gender expression and respondent gender identity on PT at the individual level. In the main effect model, message gender expression and respondent gender identity were regressed on PT with LGBT image perception and individual characteristics (age, sexual orientation, race, ethnicity, income, and smoker status) as covariates. Next, we added the interaction term of message gender expression and respondent gender identity in the model. The analysis was conducted using R.

## Results

### Study 1: Image study

Table 2 (study 1) summarizes characteristics of 461 SGM young adults as participants.

**Table 2:** Characteristics of participants

	Study 1 (*n* = 461)			Study 2 (*n* = 1190)		
	*M* (*SD*)	*n*	%	*M* (*SD*)	*n*	%
Age	24.9 (3.3)			24.6 (3.3)		
Gender identity						
Gender-expansive		125	27.1		327	27.5
Trans feminine		10	2.2		25	2.1
Trans masculine		27	5.9		55	4.6
Cisgender woman		152	33.0		389	32.7
Cisgender man		147	31.9		394	33.1
Sexual orientation						
Gay or lesbian		72	15.6		156	13.1
Bisexual		198	43.0		416	35.0
Another^a^		191	41.4		618	51.9
Race						
White		277	60.1		817	68.7
Black/African American		57	12.4		105	8.8
Asian		25	5.4		92	7.7
American Indian or Alaska Native		3	.7		10	.8
Mixed race		63	13.7		143	12.0
Another		36	7.8		23	1.9
Ethnicity^b^						
Hispanic		—	—		170	14.3
Non-Hispanic		—	—		1020	85.7
Education						
High school or less		87	18.9		233	19.6
Some college or associate degree		198	43.0		487	40.9
Bachelor’s degree		151	32.8		386	32.4
Graduate degree		25	5.4		84	7.1
Income						
<$20 000		91	19.7		230	19.3
$20 000 to $49 999		167	36.2		367	30.9
$50 000 to $74 999		86	18.7		238	20.0
$75 000 to $99 999		54	11.7		148	12.5
$100 000 or more		63	13.7		206	17.3
Marital status						
Married or living as married		75	16.3		—	—
Single		386	83.7		—	—
Smoker status						
Smoker		42	9.1		215	18.1
Social or occasional smoker		45	9.8		—	—
Ex-smoker		42	9.1		—	—
Someone who tried smoking		47	10.2		—	—
Non-smoker		285	61.8		975	81.9

a Another includes three straight/heterosexual gender-expansive participants in studies 1 and 2 straight/heterosexual gender-expansive participants in study 2.

b Ethnicity is missing in study 1 because it was not collected from participants in study 1.

For the main effect of image gender expression, there was no difference in PT between transgender/nonbinary expressions and any other gender expressions (*P*s > .05), but feminine expressions and multiple gender expressions led to significantly higher PT than masculine expressions (*B* = .16, *SE* = .08, *P* < .05; *B* = .23, *SE* = .10, *P* < .05). For the main effect of individual gender identity, when compared to cisgender SMM, gender-expansive people (*B* = .41, *SE* = .10, *P* < .001), trans masculine people (*B* = .35, *SE* = .16, *P* < .05), and cisgender SMW (*B* = .36, *SE* = .09, *P* < .001) consistently rated significantly higher PT. There was no difference between cisgender SMM and trans feminine people (*B* = .29, *SE* = .25, *P* > .05).

**Figure 1:**
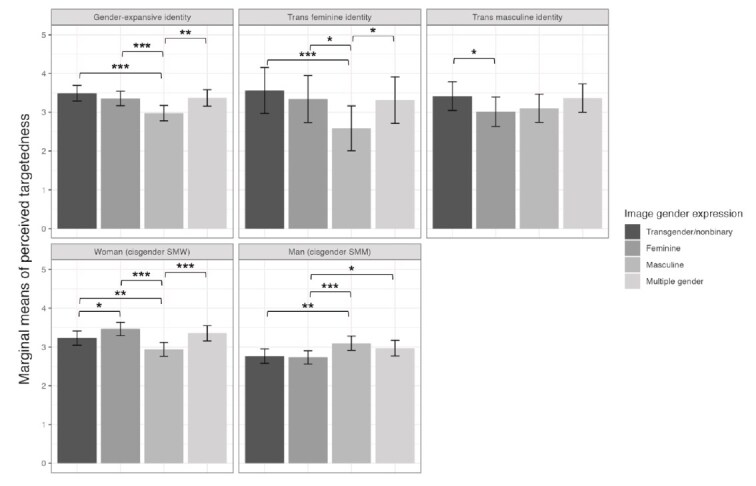
Comparisons of perceived targetedness among image gender expressions by gender identity (study 1). ^*^P < .05, ^**^P < .01, ^***^P < .001. The error bars represent 95% confidence interval.

Figure 1 presents the comparisons of PT among image gender expressions by gender identity. **H1** predicted that gender-expansive individuals would report higher PT when viewing messages using transgender/nonbinary expressions than messages using other gender expressions. Partially consistent with H1, we found that gender-expansive participants reported higher PT when viewing transgender/nonbinary expressions relative to masculine expressions (*B* = .51, *SE* = .11, *P* < .001), but the difference was not significant in comparison with feminine expressions (*B* = .13, *SE* = .11, *P* > .05). Additionally, compared to masculine expressions, the gender-expansive group rated higher PT when viewing feminine expressions (*B* = .38, *SE* = .11, *P* < .001) and multiple gender expression (*B* = .39, *SE* = .12, *P* < .01).

**H2** predicted that cisgender SMW would report higher PT when viewing messages using feminine expressions than messages using other gender expressions. Consistent with H2, cisgender SMW reported higher PT when viewing feminine expressions relative to transgender/nonbinary expressions (*B* = .24, *SE* = .10, *P* < .05) and masculine expressions (*B* = .52, *SE* = .10, *P* < .001). Compared to masculine expressions, transgender/nonbinary expressions (*B* = .29, *SE* = .10, *P* < .01) and multiple gender expressions (*B* = .41, *SE* = .12, *P* < .001) also led to higher PT within the woman group.

**H3** predicted that cisgender SMM would report higher PT when viewing messages using masculine expressions than messages using other gender expressions. Consistent with H3, cisgender SMM reported higher PT when viewing masculine expressions relative to transgender/nonbinary expressions (*B* = .33, *SE* = .10, *P* < .01) and feminine expressions (*B* = .36, *SE* = .10, *P* < .001). The cis SMM group also reported higher PT when viewing multiple gender expressions relative to feminine expressions (*B* = .24, *SE* = .11, *P* < .05).

**RQ1** asked whether trans feminine individuals would report higher PT when viewing messages using transgender/nonbinary expressions and feminine expressions compared with messages using other gender expressions. Results showed that compared to masculine expressions, trans feminine participants reported higher PT when viewing transgender/nonbinary expressions (*B* = .98, *SE* = .30, *P* < .001), feminine expressions (*B* = .76, *SE* = .30, *P* < .05), and multiple gender expressions (*B* = .73, *SE* = .30, *P* < .05). There was no significant difference between transgender/nonbinary and feminine expressions (*B* = .22, *SE* = .31, *P* > .05).

**RQ2** asked whether trans masculine individuals would report higher PT when viewing messages using transgender/nonbinary expressions and masculine expressions compared with messages using other gender expressions. We found that trans masculine participants only reported higher PT when viewing transgender/nonbinary expressions relative to feminine expressions (*B* = .40, *SE* = .20, *P* < .05). Other pairwise comparisons showed no significant differences (*P*s > .05).

**RQ3** asked whether SGM individuals would rate the PT of messages using multiple gender expressions similarly as the PT of messages using gender expressions matching individuals’ gender identity. Results showed that there was no significant difference between multiple gender and transgender/nonbinary expressions in the gender-expansive group, trans feminine group, or trans masculine group (*P*s > .05). The difference between multiple gender expressions and matched gender expressions was also insignificant in the cisgender SMW group or in the cisgender SMM group (*P*s > .05).

### Study 2: Anti-smoking message study

Table 2 (study 2) summarizes characteristics of 1190 SGM young adults as participants.

There was a significant main effect of message gender expression such that participants rated the PT of messages representing multiple gender expressions significantly higher than those messages with masculine expressions (*B* = .17, *SE* = .07, *P* < .05). The differences among other message gender expressions were not significant (*P*s > .05). There was a significant main effect of individual gender identity such that cisgender SMW rated PT of messages overall significantly higher than cis SMM (*B* = .16, *SE* = .06, *P* < .05). The differences between other comparisons by participant gender identity were not significant (*P*s > .05).

**Figure 2:**
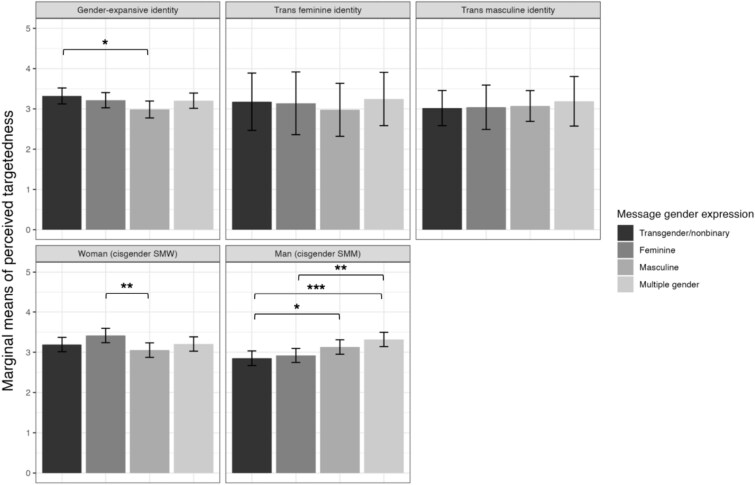
Comparisons of perceived targetedness among message gender expressions by gender identity (study 2). ^*^P < .05, ^**^P < .01, ^***^P < .001. The error bars represent 95% confidence interval.

Figure 2 presents the comparisons of PT among message gender expressions by gender identity. Partially consistent with **H1**, gender-expansive participants reported higher PT when viewing messages with transgender/nonbinary expressions relative to messages with masculine expressions (*B* = .34, *SE* = .15, *P* < .05), but the difference was not significant in comparison with viewing messages with feminine expressions (*B* = .11, *SE* = .14, *P* > .05).

Results showed that cisgender SMW reported higher PT when viewing messages with feminine expressions relative to masculine expressions (*B* = .36, *SE* = .13, *P* < .01), but the difference was not significant when compared to viewing messages with transgender/nonbinary expressions (*B* = .22, *SE* = .13, *P* > .05). **H2** was partially supported.

Partially consistent with **H3**, cisgender SMM reported higher PT when viewing messages with masculine expressions relative to transgender/nonbinary expression (*B* = .28, *SE* = .13, *P* < .05), but the difference was not significant when compared to messages with feminine expressions (*B* = .21, *SE* = .13, *P* > .05). Additionally, viewing messages with multiple gender expressions led to significantly higher PT relative to messages with transgender/nonbinary expressions (*B* = .47, *SE* = .13, *P* < .001) and feminine expressions (*B* = .40, *SE* = .13, *P* < .01).

Regarding **RQ1** and **RQ2**, there was no significant difference in PT between any message gender expressions within the trans feminine group or the trans masculine group (*P*s > .05). Regarding **RQ3**, we found no significant difference between multiple gender expressions and gender expressions matching individuals’ gender identity (*P*s > .05).

### Sensitivity analysis

Given that sexual expressions may also influence PT, we conducted several sensitivity analyses to assess the robustness of the results. After controlling for two sexual expressions, including perception of gay or lesbian individuals and perception of bisexual individuals, we found that the results remained largely unchanged across both studies. See *Supplementary Materials: Sensitivity Analysis*.

## Discussion

Through two experiments, we examined how gender expressions influenced the effects of anti-smoking campaign messages on PT. Our results suggest that messages using images portraying gender expressions that match the message receiver’s gender identity lead to higher PT than those using unmatched gender expressions. This finding is consistent with our inference that the distinctive characteristic of a social group impacts within-group individuals’ PT. The results align with the literature on matching effect, arguing that messages matching receivers’ characteristics, beliefs, and values are more effective than unmatched messages.^38^ Nevertheless, the matching effects are less clear in study 2. It is possible that the statistical power of study 2 was lower due to its between-subjects design and the relatively small sample sizes for the subgroup analyses.

We also found that gender-expansive individuals and cisgender SMW rated messages using masculine expressions as having low PT. In contrast, cisgender SMM constantly reported low PT when receiving messages using transgender/nonbinary expressions or feminine expressions. This finding is consistent with the results of a previous study that compared with women and nonbinary individuals, men had more negative attitudes toward messages using images of androgynous characters.^39^ Additionally, gender may be a more salient characteristic of gender-expansive and transgender individuals relative to cisgender individuals within the SGM community.^40^ Therefore, it is likely that gender-based tailored messages are more effective to gender-expansive and transgender individuals than cisgender SMW and SMM.

Further, we found that anti-smoking messages using multiple gender expressions were associated with higher PT among participants than those using unmatched gender expressions. This finding suggests that inclusive gender expressions presented in a message may resonate with message receivers because they can identify one or more gender expressions consistent with their gender identity. It is not surprising that the matched gender expression was effective, as gender identity is a distinctive characteristic of the SGM population. It also can be inferred that SGM individuals prefer diverse portrayals of SGM individuals in messages as they have higher PT when receiving these messages.^16^

Although there was a smaller number of transgender participants, the results showed a different pattern of PT between trans masculine and trans feminine participants. Trans feminine participants rated the PT of messages portraying transgender/nonbinary expression and feminine expressions similarly. Still, they rated the PT of messages with masculine expressions lower than transgender/nonbinary, feminine, and multiple gender expressions. However, we did not observe such a trend in the trans masculine group. We surmise that other than transgender/nonbinary expression, using messages portraying feminine expressions might be effective in targeting trans feminine audiences.

Gender-based tailored messages do not always lead to positive outcomes among SGM individuals as expected. In recent years, consumer brands gradually support transgender and nonbinary portrayals in advertising, but SGM individuals are concerned about the profit-driven motives of campaigns.^41^ Those tailored messages may cause a backlash among SGM individuals if they perceive the representation of transgender and nonbinary is not authentic and believe the messaging is only for the brands’ benefit.^42^ The importance of message authenticity is also emphasized by staff and leaders of community-based organizations serving SGM populations.^31^ Health communication practitioners must carefully consider how audiences perceive the authenticity of SGM-tailored messages.

This study found that PT warranted more attention in tailoring anti-smoking messages for SGM audiences. Our results demonstrate that segmenting SGM individuals and tailoring messages based on gender identity is crucial to increasing PT among diverse SGM young adult audiences. Our results also have practical implications for practitioners (especially those focusing on SGM health) who want to improve the health of minority groups and reduce health disparities. By respecting distinctive characteristics among minority groups and tailoring messages based on those characteristics, message designers are more likely to create effective culturally tailored messages. For example, messages tailored for trans feminine individuals can use messengers who identify as trans feminine (instead of other SGM identities) and languages that resonate with the trans feminine community.^13^

There are several limitations to this study. First, the small sample of transgender participants limited our understanding of gender expression effects. Second, participants may have perceived a different gender expression of specific images than intended. We attempted to address this by using multiple images within each gender expression condition and partially confirmed the effective manipulation of gender expression (Tables S2 and S6). Third, the interplay between gender identity, gender expression, sexual identity, and sexual attraction require future investigation using more nuanced measures of PT and intersectional analysis of multiple identities. Fourth, given that an individual’s gender expression may be distinct from their gender identity (eg, a cisgender SMW may identify with masculine expression), we may have underestimated the matching effects, as we did not directly measure individual gender expression. Fifth, although higher PT is associated with persuasion outcomes (eg, positive attitude toward a message^29^^,^^43^), this relationship remains unexplored in the current context. Sixth, the effects of subgroup-specific health messages in addition to images alone (eg, using an image paired with a message tailored for lesbian women) require further study. Finally, our findings may not be generalizable to U.S. SGM young adults. Replication research using a nationally representative sample is needed.

In conclusion, this study examines the role of gender in tailoring anti-smoking messages for SGM young adults and highlights the importance of measuring PT in relevant research. Our results suggest that SGM young adults perceive high PT when receiving campaign messages using gender expressions consistent with their respective gender identity or featuring multiple gender expressions. We should not view the SGM community as a monolith in smoking interventions and need to tailor messages to different gender groups within the community.

## Supplementary Material

Supplementary_Materials_ntag026

## Data Availability

A restricted dataset may be requested from the corresponding author (Qijia Ye: qijia.ye@asc.upenn.edu) and should include a plan for its use. All data sharing will comply with local, state, and federal laws and regulations and may be subject to appropriate human subjects institutional review board approvals.
