# Supplementary material for: Does gender expression matter in tailoring anti-smoking messages for sexual and gender minority young adults?
Source: Nicotine Tob Res. 2026 Feb 13;28(7):1217–27. doi: 10.1093/ntr/ntag026 (PMC13286645; doi:10.1093/ntr/ntag026)
Supplement: Supplementary_Materials_ntag026 [file supplementary_materials_ntag026.docx]

**Supplementary Materials: Study 1**

**Table S1**

Characteristics of Participants by Gender Identity (Study 1)

|  | Gender-expansive  (*N* = 125) | | | Trans feminine  (*N* = 10) | | | Trans masculine  (*N* = 27) | | | Cisgender SMW  (*N* = 152) | | | | Cisgender SMM  (*N* = 147) | | |
| --- | --- | --- | --- | --- | --- | --- | --- | --- | --- | --- | --- | --- | --- | --- | --- | --- |
|  | *M* (*SD*) | *n* | % | *M* (*SD*) | *n* | % | *M* (*SD*) | *n* | % | *M* (*SD*) | *n* | % | *M* (*SD*) | | *n* | % |
| Age | 23.8  (3.2) |  |  | 26.8  (2.1) |  |  | 24.3  (3.2) |  |  | 25.1 (3.3) |  |  | 25.5 (3.2) | |  |  |
| Sexual orientation |  |  |  |  |  |  |  |  |  |  |  |  |  | |  |  |
| Gay or lesbian |  | 15 | 12.0 |  | 0 | 0 |  | 4 | 14.8 |  | 17 | 11.2 |  | | 36 | 24.5 |
| Bisexual |  | 28 | 22.4 |  | 3 | 30.0 |  | 12 | 44.4 |  | 78 | 51.3 |  | | 77 | 52.4 |
| Other^a^ |  | 82 | 65.6 |  | 7 | 70.0 |  | 11 | 40.7 |  | 57 | 37.5 |  | | 34 | 23.1 |
| Race |  |  |  |  |  |  |  |  |  |  |  |  |  | |  |  |
| White |  | 83 | 66.4 |  | 6 | 60.0 |  | 17 | 63.0 |  | 89 | 58.6 |  | | 82 | 55.8 |
| Black/African American |  | 8 | 6.4 |  | 1 | 10.0 |  | 3 | 11.1 |  | 21 | 13.8 |  | | 24 | 16.3 |
| Other^b^ |  | 32 | 25.6 |  | 2 | 20.0 |  | 7 | 25.9 |  | 42 | 27.6 |  | | 39 | 26.5 |
| Not to answer |  | 2 | 1.6 |  | 1 | 10.0 |  | 0 | 0 |  | 0 | 0 |  | | 2 | 1.4 |
| Education |  |  |  |  |  |  |  |  |  |  |  |  |  | |  |  |
| High school or less |  | 28 | 22.4 |  | 1 | 10.0 |  | 6 | 22.2 |  | 24 | 15.8 |  | | 28 | 19.0 |
| Some college or associate degree |  | 56 | 44.8 |  | 7 | 70.0 |  | 12 | 44.4 |  | 66 | 43.4 |  | | 57 | 38.8 |
| Bachelor’s degree |  | 36 | 18.8 |  | 2 | 20.0 |  | 8 | 29.6 |  | 50 | 32.9 |  | | 55 | 37.4 |
| Graduate degree |  | 5 | 4.0 |  | 0 | 0 |  | 1 | 3.7 |  | 12 | 7.9 |  | | 7 | 4.8 |
| Income |  |  |  |  |  |  |  |  |  |  |  |  |  | |  |  |
| Less than $20, 000 |  | 34 | 27.2 |  | 4 | 40.0 |  | 4 | 14.8 |  | 21 | 13.8 |  | | 28 | 19.0 |
| $ 20,000 to $ 49,999 |  | 47 | 37.6 |  | 4 | 40.0 |  | 7 | 25.9 |  | 62 | 40.8 |  | | 47 | 32.0 |
| $ 50,000 to $ 74,999 |  | 24 | 19.2 |  | 2 | 20.0 |  | 6 | 22.2 |  | 26 | 17.1 |  | | 28 | 19.0 |
| $ 75,000 to $ 99,999 |  | 9 | 7.2 |  | 0 | 0 |  | 5 | 18.5 |  | 21 | 13.8 |  | | 19 | 12.9 |
| $ 100,000 or more |  | 11 | 8.8 |  | 0 | 0 |  | 5 | 18.5 |  | 22 | 14.5 |  | | 25 | 17.0 |
| Marital status |  |  |  |  |  |  |  |  |  |  |  |  |  | |  |  |
| Married or living as married |  | 18 | 14.4 |  | 2 | 20.0 |  | 7 | 25.9 |  | 30 | 19.7 |  | | 18 | 12.2 |
| Single |  | 107 | 85.6 |  | 8 | 80.0 |  | 20 | 74.1 |  | 122 | 80.3 |  | | 129 | 87.8 |
| Smoker status |  |  |  |  |  |  |  |  |  |  |  |  |  | |  |  |
| Smoker |  | 9 | 7.2 |  | 1 | 10.0 |  | 5 | 18.5 |  | 13 | 8.6 |  | | 14 | 9.5 |
| Social or occasional smoker |  | 14 | 11.2 |  | 0 | 0 |  | 1 | 3.7 |  | 15 | 9.9 |  | | 15 | 10.2 |
| Ex-smoker |  | 8 | 6.4 |  | 2 | 20.0 |  | 1 | 3.7 |  | 19 | 12.5 |  | | 12 | 8.2 |
| Someone who tried smoking |  | 15 | 12.0 |  | 0 | 0.0 |  | 4 | 14.8 |  | 13 | 8.6 |  | | 15 | 10.2 |
| Non-smoker |  | 79 | 63.2 |  | 7 | 70.0 |  | 16 | 59.3 |  | 92 | 60.5 |  | | 91 | 61.9 |

^a^Other includes 3 straight/heterosexual gender-expansive participants.

^b^Other includes Asian, American Indian or Alaska Native, Middle Eastern, Arab, or Arab American, Native Hawaiian, Other Pacific Islander, Samoan, and Mixed Race.

**Table S2**

Effects of Gender Expression on Transgender Image Perception (Study 1)

|  | *OR* | 95% *CI* | |
| --- | --- | --- | --- |
|  |  | *LL* | *UL* |
| Intercept | 0.22 | 0.04 | 1.24 |
| Gender expression (Referent: Transgender/nonbinary expressions) |  |  |  |
| Feminine expressions | 0.06^***^ | 0.02 | 0.20 |
| Masculine expressions | 0.03^***^ | 0.01 | 0.09 |
| Multiple gender expressions | 0.25^+^ | 0.06 | 1.03 |
| Respondent gender identity (Referent: Gender-expansive/transgender identity) |  |  |  |
| Woman (cisgender SMW) | 0.36^***^ | 0.23 | 0.57 |
| Man (cisgender SMM) | 0.36^***^ | 0.23 | 0.58 |
| Number of characters | 1.12 | 0.78 | 1.60 |
| Age | 1.04 | 0.99 | 1.10 |
| Sexual orientation |  |  |  |
| Gay or lesbian | 0.70 | 0.41 | 1.20 |
| Other | 1.67 | 1.12 | 2.50 |
| Race |  |  |  |
| Black | 0.48 | 0.27 | 0.85 |
| Other | 0.94 | 0.63 | 1.41 |
| Smoker status |  |  |  |
| Occasional smoker | 0.76 | 0.34 | 1.73 |
| Ex-smoker | 0.73 | 0.32 | 1.66 |
| Someone who tried smoking | 0.64 | 0.29 | 1.43 |
| Non-smoker | 1.22 | 0.66 | 2.25 |
| Random components: Variance |  |  |  |
| Image level | 2.84 |  |  |
| Individual level | 1.87 |  |  |
| *N* (total observations) | 3687 |  |  |
| *N* (image) | 80 |  |  |
| *N* (individual) | 461 |  |  |

*Note*. Transgender image perception is coded as a binary outcome: 0 = No, 1 = Yes. Results are obtained from the cross-classified logistic regression model conducted in R. Gender expression: Transgender/nonbinary expressions is reference category. Respondent gender identity: Gender-expansive/transgender identity is reference category. Sexual orientation: Bisexual is reference category. Race: White is reference category. Smoker status: Smoker is reference category. Age and number of characters are treated as continuous variables. ^+^ *p* < .08, ^*^ *p* < .05, ^**^ *p* < .01, ^***^ *p* < .001.

**Table S3.1**

Effects of Image Gender Expression and Respondent Gender Identity on Perceived Targetedness (Study 1)

|  | Main Effect | | Interaction Effect | |
| --- | --- | --- | --- | --- |
|  | *B* | *SE* | *B* | *SE* |
| Intercept | 2.70^***^ | 0.34 | 2.94^***^ | 0.35 |
| Gender expression (Referent: Transgender/nonbinary expressions) |  |  |  |  |
| Feminine expressions | 0.00 | 0.08 | −0.13 | 0.11 |
| Masculine expressions | −0.15^+^ | 0.08 | −0.51^***^ | 0.11 |
| Multiple gender expressions | 0.08 | 0.10 | −0.12 | 0.13 |
| Respondent gender identity (Referent: Gender-expansive identity) |  |  |  |  |
| Trans feminine | −0.12 | 0.25 | 0.07 | 0.31 |
| Trans masculine | −0.06 | 0.16 | −0.08 | 0.20 |
| Woman (cisgender SMW) | −0.05 | 0.09 | −0.26^*^ | 0.12 |
| Man (cisgender SMM) | −0.41^***^ | 0.10 | −0.72^***^ | 0.12 |
| LGBT image perception | 0.63^***^ | 0.04 | 0.61^***^ | 0.04 |
| Number of characters | 0.06^*^ | 0.03 | 0.06^*^ | 0.03 |
| Age | 0.01 | 0.01 | 0.01 | 0.01 |
| Sexual orientation |  |  |  |  |
| Gay or lesbian | 0.32^**^ | 0.10 | 0.32^**^ | 0.10 |
| Other | −0.05 | 0.08 | −0.05 | 0.08 |
| Race |  |  |  |  |
| Black | −0.13 | 0.11 | −0.13 | 0.11 |
| Other | −0.06 | 0.08 | −0.06 | 0.08 |
| Education |  |  |  |  |
| Some college | −0.01 | 0.10 | −0.01 | 0.10 |
| Bachelor’s degree | 0.18 | 0.11 | 0.19^+^ | 0.11 |
| Graduate degree | −0.03 | 0.18 | 0.01 | 0.18 |
| Income | −0.00 | 0.02 | −0.00 | 0.02 |
| Marital status | 0.01 | 0.10 | 0.00 | 0.10 |
| Smoker status |  |  |  |  |
| Occasional smoker | −0.26 | 0.16 | −0.27 | 0.16 |
| Ex-smoker | −0.38^*^ | 0.16 | −0.39^*^ | 0.16 |
| Someone who tried smoking | −0.11 | 0.16 | −0.13 | 0.16 |
| Non-smoker | −0.26^*^ | 0.13 | −0.28^*^ | 0.13 |
| Interaction |  |  |  |  |
| Trans feminine × Feminine expressions | ⎯ | ⎯ | −0.09 | 0.31 |
| Trans feminine × Masculine expressions | ⎯ | ⎯ | −0.46 | 0.30 |
| Trans feminine × Multiple gender expressions | ⎯ | ⎯ | −0.13 | 0.31 |
| Trans masculine × Feminine expressions | ⎯ | ⎯ | −0.27 | 0.21 |
| Trans masculine × Masculine expressions | ⎯ | ⎯ | 0.20 | 0.20 |
| Trans masculine × Multiple gender expressions | ⎯ | ⎯ | 0.07 | 0.20 |
| Woman (cisgender SMW) × Feminine expressions | ⎯ | ⎯ | 0.37^**^ | 0.11 |
| Woman (cisgender SMW) × Masculine expressions | ⎯ | ⎯ | 0.23^+^ | 0.12 |
| Woman (cisgender SMW) × Multiple gender expressions | ⎯ | ⎯ | 0.25^*^ | 0.12 |
| Man (cisgender SMM) × Feminine expressions | ⎯ | ⎯ | 0.10 | 0.11 |
| Man (cisgender SMM) × Masculine expressions | ⎯ | ⎯ | 0.84^***^ | 0.12 |
| Man (cisgender SMM) × Multiple gender expressions | ⎯ | ⎯ | 0.33^**^ | 0.12 |
| Random components: Variance |  |  |  |  |
| Image level | 0.04 |  | 0.04 |  |
| Individual level | 0.43 |  | 0.44 |  |
| Residual | 0.83 |  | 0.81 |  |
| *N* (total observations) | 3685 |  | 3685 |  |
| *N* (image) | 80 |  | 80 |  |
| *N* (individual) | 461 |  | 461 |  |

*Note*. All coefficients are unstandardized. Gender expression: Transgender/nonbinary expressions is reference category. Respondent gender identity: Gender-expansive identity is reference category. LGBT image perception: No is reference category. Sexual orientation: Bisexual is reference category. Race: White is reference category. Education: High school or less is reference category. Marital status: Married or living as married is reference category. Smoker status: Smoker is reference category. Age, income, and number of characters are treated as continuous variables. ^+^ *p* < .08, ^*^ *p* < .05, ^**^ *p* < .01, ^***^ *p* < .001.

**Table S3.2**

Effects of Image Gender Expression and Respondent Gender Identity on Perceived Targetedness (Study 1)

|  | Main Effect | | Interaction Effect | |
| --- | --- | --- | --- | --- |
|  | *B* | *SE* | *B* | *SE* |
| Intercept | 2.58^***^ | 0.43 | 3.01^***^ | 0.47 |
| Gender expression (Referent: Transgender/nonbinary expressions) |  |  |  |  |
| Feminine expressions | 0.00 | 0.08 | −0.22 | 0.31 |
| Masculine expressions | −0.15^+^ | 0.08 | −0.98^***^ | 0.30 |
| Multiple gender expressions | 0.08 | 0.10 | −0.25 | 0.31 |
| Respondent gender identity (Referent: Trans feminine identity) |  |  |  |  |
| Gender-expansive | 0.12 | 0.25 | −0.07 | 0.31 |
| Trans masculine | 0.06 | 0.28 | −0.15 | 0.35 |
| Woman (cisgender SMW) | 0.07 | 0.24 | −0.33 | 0.31 |
| Man (cisgender SMM) | −0.29 | 0.25 | −0.80^*^ | 0.31 |
| LGBT image perception | 0.63^***^ | 0.04 | 0.61^***^ | 0.04 |
| Number of characters | 0.06^*^ | 0.03 | 0.06^*^ | 0.03 |
| Age | 0.01 | 0.01 | 0.01 | 0.01 |
| Sexual orientation |  |  |  |  |
| Gay or lesbian | 0.32^**^ | 0.10 | 0.32^**^ | 0.10 |
| Other | −0.05 | 0.08 | −0.05 | 0.08 |
| Race |  |  |  |  |
| Black | −0.13 | 0.11 | −0.13 | 0.11 |
| Other | −0.06 | 0.08 | −0.06 | 0.08 |
| Education |  |  |  |  |
| Some college | −0.01 | 0.10 | −0.01 | 0.10 |
| Bachelor’s degree | 0.18 | 0.11 | 0.19^+^ | 0.11 |
| Graduate degree | −0.03 | 0.18 | 0.01 | 0.18 |
| Income | −0.00 | 0.02 | −0.00 | 0.02 |
| Marital status | 0.01 | 0.10 | 0.00 | 0.10 |
| Smoker status |  |  |  |  |
| Occasional smoker | −0.26 | 0.16 | −0.27 | 0.16 |
| Ex-smoker | −0.38^*^ | 0.16 | −0.39^*^ | 0.16 |
| Someone who tried smoking | −0.11 | 0.16 | −0.13 | 0.16 |
| Non-smoker | −0.26^*^ | 0.13 | −0.28^*^ | 0.13 |
| Interaction |  |  |  |  |
| Gender-expansive × Feminine expressions | ⎯ | ⎯ | 0.09 | 0.31 |
| Gender-expansive × Masculine expressions | ⎯ | ⎯ | 0.46 | 0.30 |
| Gender-expansive × Multiple gender expressions | ⎯ | ⎯ | 0.13 | 0.31 |
| Trans masculine × Feminine expressions | ⎯ | ⎯ | −0.18 | 0.36 |
| Trans masculine × Masculine expressions | ⎯ | ⎯ | 0.66^+^ | 0.34 |
| Trans masculine × Multiple gender expressions | ⎯ | ⎯ | 0.20 | 0.35 |
| Woman (cisgender SMW) × Feminine expressions | ⎯ | ⎯ | 0.46 | 0.31 |
| Woman (cisgender SMW) × Masculine expressions | ⎯ | ⎯ | 0.69^*^ | 0.30 |
| Woman (cisgender SMW) × Multiple gender expressions | ⎯ | ⎯ | 0.38 | 0.31 |
| Man (cisgender SMM) × Feminine expressions | ⎯ | ⎯ | 0.19 | 0.31 |
| Man (cisgender SMM) × Masculine expressions | ⎯ | ⎯ | 1.31^***^ | 0.30 |
| Man (cisgender SMM) × Multiple gender expressions | ⎯ | ⎯ | 0.46 | 0.31 |
| Random components: Variance |  |  |  |  |
| Image level | 0.04 |  | 0.04 |  |
| Individual level | 0.43 |  | 0.44 |  |
| Residual | 0.83 |  | 0.81 |  |
| *N* (total observations) | 3685 |  | 3685 |  |
| *N* (image) | 80 |  | 80 |  |
| *N* (individual) | 461 |  | 461 |  |

*Note*. All coefficients are unstandardized. Gender expression: Transgender/nonbinary expressions is reference category. Respondent gender identity: Trans feminine identity is reference category. LGBT image perception: No is reference category. Sexual orientation: Bisexual is reference category. Race: White is reference category. Education: High school or less is reference category. Marital status: Married or living as married is reference category. Smoker status: Smoker is reference category. Age, income, and number of characters are treated as continuous variables. ^+^ *p* < .08, ^*^ *p* < .05, ^**^ *p* < .01, ^***^ *p* < .001.

**Table S3.3**

Effects of Image Gender Expression and Respondent Gender Identity on Perceived Targetedness (Study 1)

|  | Main Effect | | Interaction Effect | |
| --- | --- | --- | --- | --- |
|  | *B* | *SE* | *B* | *SE* |
| Intercept | 2.64^***^ | 0.37 | 2.86^***^ | 0.38 |
| Gender expression (Referent: Transgender/nonbinary expressions) |  |  |  |  |
| Feminine expressions | 0.00 | 0.08 | −0.40^*^ | 0.20 |
| Masculine expressions | −0.15^+^ | 0.08 | −0.31 | 0.19 |
| Multiple gender expressions | 0.08 | 0.10 | −0.05 | 0.20 |
| Respondent gender identity (Referent: Trans masculine identity) |  |  |  |  |
| Gender-expansive | 0.06 | 0.16 | 0.08 | 0.20 |
| Trans feminine | −0.06 | 0.28 | 0.15 | 0.35 |
| Woman (cisgender SMW) | 0.02 | 0.15 | −0.19 | 0.20 |
| Man (cisgender SMM) | −0.35^*^ | 0.16 | −0.65^**^ | 0.20 |
| LGBT image perception | 0.63^***^ | 0.04 | 0.61^***^ | 0.04 |
| Number of characters | 0.06^*^ | 0.03 | 0.06^*^ | 0.03 |
| Age | 0.01 | 0.01 | 0.01 | 0.01 |
| Sexual orientation |  |  |  |  |
| Gay or lesbian | 0.32^**^ | 0.10 | 0.32^**^ | 0.10 |
| Other | −0.05 | 0.08 | −0.05 | 0.08 |
| Race |  |  |  |  |
| Black | −0.13 | 0.11 | −0.13 | 0.11 |
| Other | −0.06 | 0.08 | −0.06 | 0.08 |
| Education |  |  |  |  |
| Some college | −0.01 | 0.10 | −0.01 | 0.10 |
| Bachelor’s degree | 0.18 | 0.11 | 0.19^+^ | 0.11 |
| Graduate degree | −0.03 | 0.18 | 0.01 | 0.18 |
| Income | −0.00 | 0.02 | −0.00 | 0.02 |
| Marital status | 0.01 | 0.10 | 0.00 | 0.10 |
| Smoker status |  |  |  |  |
| Occasional smoker | −0.26 | 0.16 | −0.27 | 0.16 |
| Ex-smoker | −0.38^*^ | 0.16 | −0.39^*^ | 0.16 |
| Someone who tried smoking | −0.11 | 0.16 | −0.13 | 0.16 |
| Non-smoker | −0.26^*^ | 0.13 | −0.28^*^ | 0.13 |
| Interaction |  |  |  |  |
| Gender-expansive × Feminine expressions | ⎯ | ⎯ | 0.27 | 0.21 |
| Gender-expansive × Masculine expressions | ⎯ | ⎯ | −0.20 | 0.20 |
| Gender-expansive × Multiple gender expressions | ⎯ | ⎯ | −0.07 | 0.20 |
| Trans feminine × Feminine expressions | ⎯ | ⎯ | 0.18 | 0.36 |
| Trans feminine × Masculine expressions | ⎯ | ⎯ | −0.66^+^ | 0.34 |
| Trans feminine × Multiple gender expressions | ⎯ | ⎯ | −0.20 | 0.35 |
| Woman (cisgender SMW) × Feminine expressions | ⎯ | ⎯ | 0.64^**^ | 0.21 |
| Woman (cisgender SMW) × Masculine expressions | ⎯ | ⎯ | 0.03 | 0.20 |
| Woman (cisgender SMW) × Multiple gender expressions | ⎯ | ⎯ | 0.18 | 0.19 |
| Man (cisgender SMM) × Feminine expressions | ⎯ | ⎯ | 0.37^+^ | 0.21 |
| Man (cisgender SMM) × Masculine expressions | ⎯ | ⎯ | 0.64^**^ | 0.20 |
| Man (cisgender SMM) × Multiple gender expressions | ⎯ | ⎯ | 0.26 | 0.20 |
| Random components: Variance |  |  |  |  |
| Image level | 0.04 |  | 0.04 |  |
| Individual level | 0.43 |  | 0.44 |  |
| Residual | 0.83 |  | 0.81 |  |
| *N* (total observations) | 3685 |  | 3685 |  |
| *N* (image) | 80 |  | 80 |  |
| *N* (individual) | 461 |  | 461 |  |

*Note*. All coefficients are unstandardized. Gender expression: Transgender/nonbinary expressions is reference category. Respondent gender identity: Trans masculine identity is reference category. LGBT image perception: No is reference category. Sexual orientation: Bisexual is reference category. Race: White is reference category. Education: High school or less is reference category. Marital status: Married or living as married is reference category. Smoker status: Smoker is reference category. Age, income, and number of characters are treated as continuous variables. ^+^ *p* < .08, ^*^ *p* < .05, ^**^ *p* < .01, ^***^ *p* < .001.

**Table S3.4**

Effects of Image Gender Expression and Respondent Gender Identity on Perceived Targetedness (Study 1)

|  | Main Effect | | Interaction Effect | |
| --- | --- | --- | --- | --- |
|  | *B* | *SE* | *B* | *SE* |
| Intercept | 2.66^***^ | 0.35 | 2.91^***^ | 0.36 |
| Gender expression (Referent: Feminine expressions) |  |  |  |  |
| Transgender/nonbinary expressions | −0.00 | 0.08 | −0.24^*^ | 0.10 |
| Masculine expressions | −0.16^*^ | 0.08 | −0.52^***^ | 0.10 |
| Multiple gender expressions | 0.07 | 0.09 | −0.11 | 0.11 |
| Respondent gender identity (Referent: Woman (cisgender SMW)) |  |  |  |  |
| Gender-expansive | 0.05 | 0.09 | −0.11 | 0.11 |
| Trans feminine | −0.07 | 0.24 | −0.12 | 0.32 |
| Trans masculine | −0.02 | 0.15 | −0.45^*^ | 0.20 |
| Man (cisgender SMM) | −0.36^***^ | 0.09 | −0.73^***^ | 0.11 |
| LGBT image perception | 0.63^***^ | 0.04 | 0.61^***^ | 0.04 |
| Number of characters | 0.06^*^ | 0.03 | 0.06^*^ | 0.03 |
| Age | 0.01 | 0.01 | 0.01 | 0.01 |
| Sexual orientation |  |  |  |  |
| Gay or lesbian | 0.32^**^ | 0.10 | 0.32^**^ | 0.10 |
| Other | −0.05 | 0.08 | −0.05 | 0.08 |
| Race |  |  |  |  |
| Black | −0.13 | 0.11 | −0.13 | 0.11 |
| Other | −0.06 | 0.08 | −0.06 | 0.08 |
| Education |  |  |  |  |
| Some college | −0.01 | 0.10 | −0.01 | 0.10 |
| Bachelor’s degree | 0.18 | 0.11 | 0.19^+^ | 0.11 |
| Graduate degree | −0.03 | 0.18 | 0.01 | 0.18 |
| Income | −0.00 | 0.02 | −0.00 | 0.02 |
| Marital status | 0.01 | 0.10 | 0.00 | 0.10 |
| Smoker status |  |  |  |  |
| Occasional smoker | −0.26 | 0.16 | −0.27 | 0.16 |
| Ex-smoker | −0.38^*^ | 0.16 | −0.39^*^ | 0.16 |
| Someone who tried smoking | −0.11 | 0.16 | −0.13 | 0.16 |
| Non-smoker | −0.26^*^ | 0.13 | −0.28^*^ | 0.13 |
| Interaction |  |  |  |  |
| Gender-expansive × Transgender/nonbinary expressions | ⎯ | ⎯ | 0.37^**^ | 0.11 |
| Gender-expansive × Masculine expressions | ⎯ | ⎯ | 0.14 | 0.11 |
| Gender-expansive × Multiple gender expressions | ⎯ | ⎯ | 0.12 | 0.11 |
| Trans feminine × Transgender/nonbinary expressions | ⎯ | ⎯ | 0.46 | 0.31 |
| Trans feminine × Masculine expressions | ⎯ | ⎯ | −0.23 | 0.31 |
| Trans feminine × Multiple gender expressions | ⎯ | ⎯ | 0.08 | 0.32 |
| Trans masculine × Transgender/nonbinary expressions | ⎯ | ⎯ | 0.64^**^ | 0.21 |
| Trans masculine × Masculine expressions | ⎯ | ⎯ | 0.61^**^ | 0.20 |
| Trans masculine × Multiple gender expressions | ⎯ | ⎯ | 0.46^*^ | 0.20 |
| Man (cisgender SMM) × Transgender/nonbinary expressions | ⎯ | ⎯ | 0.27^*^ | 0.11 |
| Man (cisgender SMM) × Masculine expressions | ⎯ | ⎯ | 0.88^***^ | 0.11 |
| Man (cisgender SMM) × Multiple gender expressions | ⎯ | ⎯ | 0.35^**^ | 0.11 |
| Random components: Variance |  |  |  |  |
| Image level | 0.04 |  | 0.04 |  |
| Individual level | 0.43 |  | 0.44 |  |
| Residual | 0.83 |  | 0.81 |  |
| *N* (total observations) | 3685 |  | 3685 |  |
| *N* (image) | 80 |  | 80 |  |
| *N* (individual) | 461 |  | 461 |  |

*Note*. All coefficients are unstandardized. Gender expression: Feminine expressions is reference category. Respondent gender identity: Woman (cisgender SMW) identity is reference category. LGBT image perception: No is reference category. Sexual orientation: Bisexual is reference category. Race: White is reference category. Education: High school or less is reference category. Marital status: Married or living as married is reference category. Smoker status: Smoker is reference category. Age, income, and number of characters are treated as continuous variables. ^+^ *p* < .08, ^*^ *p* < .05, ^**^ *p* < .01, ^***^ *p* < .001.

**Table S3.5**

Effects of Image Gender Expression and Respondent Gender Identity on Perceived Targetedness (Study 1)

|  | Main Effect | | Interaction Effect | |
| --- | --- | --- | --- | --- |
|  | *B* | *SE* | *B* | *SE* |
| Intercept | 2.14^**^ | 0.36 | 2.54^***^ | 0.36 |
| Gender expression (Referent: Masculine expressions) |  |  |  |  |
| Transgender/nonbinary expressions | 0.15^+^ | 0.08 | −0.33^**^ | 0.10 |
| Feminine expressions | 0.16^*^ | 0.08 | −0.36^***^ | 0.10 |
| Multiple gender expressions | 0.23^*^ | 0.10 | −0.12 | 0.12 |
| Respondent gender identity (Referent: Man (cisgender SMM)) |  |  |  |  |
| Gender-expansive | 0.41^***^ | 0.10 | −0.12 | 0.12 |
| Trans feminine | 0.29 | 0.25 | −0.51 | 0.30 |
| Trans masculine | 0.35^*^ | 0.16 | 0.01 | 0.20 |
| Woman (cisgender SMW) | 0.36^***^ | 0.09 | −0.15 | 0.10 |
| LGBT image perception | 0.63^***^ | 0.04 | 0.61^***^ | 0.04 |
| Number of characters | 0.06^*^ | 0.03 | 0.06^*^ | 0.03 |
| Age | 0.01 | 0.01 | 0.01 | 0.01 |
| Sexual orientation |  |  |  |  |
| Gay or lesbian | 0.32^**^ | 0.10 | 0.32^**^ | 0.10 |
| Other | −0.05 | 0.08 | −0.05 | 0.08 |
| Race |  |  |  |  |
| Black | −0.13 | 0.11 | −0.13 | 0.11 |
| Other | −0.06 | 0.08 | −0.06 | 0.08 |
| Education |  |  |  |  |
| Some college | −0.01 | 0.10 | −0.01 | 0.10 |
| Bachelor’s degree | 0.18 | 0.11 | 0.19^+^ | 0.11 |
| Graduate degree | −0.03 | 0.18 | 0.01 | 0.18 |
| Income | −0.00 | 0.02 | −0.00 | 0.02 |
| Marital status | 0.01 | 0.10 | 0.00 | 0.10 |
| Smoker status |  |  |  |  |
| Occasional smoker | −0.26 | 0.16 | −0.27 | 0.16 |
| Ex-smoker | −0.38^*^ | 0.16 | −0.39^*^ | 0.16 |
| Someone who tried smoking | −0.11 | 0.16 | −0.13 | 0.16 |
| Non-smoker | −0.26^*^ | 0.13 | −0.28^*^ | 0.13 |
| Interaction |  |  |  |  |
| Gender-expansive × Transgender/nonbinary expressions | ⎯ | ⎯ | 0.84^***^ | 0.12 |
| Gender-expansive × Feminine expressions | ⎯ | ⎯ | 0.74^***^ | 0.11 |
| Gender-expansive × Multiple gender expressions | ⎯ | ⎯ | 0.52^***^ | 0.12 |
| Trans feminine × Transgender/nonbinary expressions | ⎯ | ⎯ | 1.31^***^ | 0.30 |
| Trans feminine × Feminine expressions | ⎯ | ⎯ | 1.12^***^ | 0.30 |
| Trans feminine × Multiple gender expressions | ⎯ | ⎯ | 0.85^**^ | 0.30 |
| Trans masculine × Transgender/nonbinary expressions | ⎯ | ⎯ | 0.64^**^ | 0.20 |
| Trans masculine × Feminine expressions | ⎯ | ⎯ | 0.27 | 0.20 |
| Trans masculine × Multiple gender expressions | ⎯ | ⎯ | 0.39^*^ | 0.19 |
| Woman (cisgender SMW) × Transgender/nonbinary expressions | ⎯ | ⎯ | 0.61^***^ | 0.11 |
| Woman (cisgender SMW) × Feminine expressions | ⎯ | ⎯ | 0.88^***^ | 0.11 |
| Woman (cisgender SMW) × Multiple gender expressions | ⎯ | ⎯ | 0.54^***^ | 0.11 |
| Random components: Variance |  |  |  |  |
| Image level | 0.04 |  | 0.04 |  |
| Individual level | 0.43 |  | 0.44 |  |
| Residual | 0.83 |  | 0.81 |  |
| *N* (total observations) | 3685 |  | 3685 |  |
| *N* (image) | 80 |  | 80 |  |
| *N* (individual) | 461 |  | 461 |  |

*Note*. All coefficients are unstandardized. Gender expression: Masculine expressions is reference category. Respondent gender identity: Man (cisgender SMM) identity is reference category. LGBT image perception: No is reference category. Sexual orientation: Bisexual is reference category. Race: White is reference category. Education: High school or less is reference category. Marital status: Married or living as married is reference category. Smoker status: Smoker is reference category. Age, income, and number of characters are treated as continuous variables. ^+^ *p* < .08, ^*^ *p* < .05, ^**^ *p* < .01, ^***^ *p* < .001.

**Table S4**

Marginal Means of Perceived Targetedness (Study 1)

| Gender expression | Gender-expansive | | | Trans feminine | | | Trans masculine | | | Cisgender SMW | | | Cisgender SMM | | |
| --- | --- | --- | --- | --- | --- | --- | --- | --- | --- | --- | --- | --- | --- | --- | --- |
|  | *M* | 95% *CI* | | *M* | 95% *CI* | | *M* | 95% *CI* | | *M* | 95% *CI* | | *M* | 95% *CI* | |
|  |  | *LL* | *UL* |  | *LL* | *UL* |  | *LL* | *UL* |  | *LL* | *UL* |  | *LL* | *UL* |
| Transgender/nonbinary expressions | 3.49 | 3.29 | 3.69 | 3.56 | 2.97 | 4.15 | 3.42 | 3.05 | 3.79 | 3.23 | 3.05 | 3.41 | 2.77 | 2.58 | 2.95 |
| Feminine expressions | 3.36 | 3.17 | 3.54 | 3.34 | 2.73 | 3.95 | 3.01 | 2.63 | 3.39 | 3.46 | 3.29 | 3.64 | 2.73 | 2.56 | 2.91 |
| Masculine expressions | 2.98 | 2.78 | 3.18 | 2.59 | 2.01 | 3.17 | 3.10 | 2.74 | 3.47 | 2.94 | 2.76 | 3.12 | 3.10 | 2.91 | 3.28 |
| Multiple gender expressions | 3.37 | 3.16 | 3.58 | 3.31 | 2.71 | 3.91 | 3.37 | 3.00 | 3.73 | 3.36 | 3.16 | 3.55 | 2.97 | 2.77 | 3.18 |

**Figure S1**

Example Images Representing Each Gender Expression (Study 1)

| Transgender/nonbinary expression | | |
| --- | --- | --- |
| 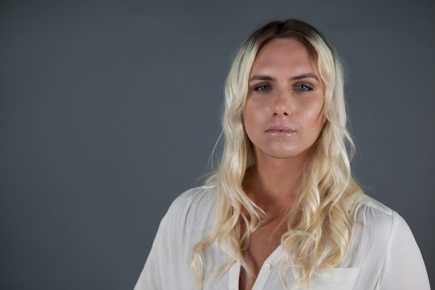 | 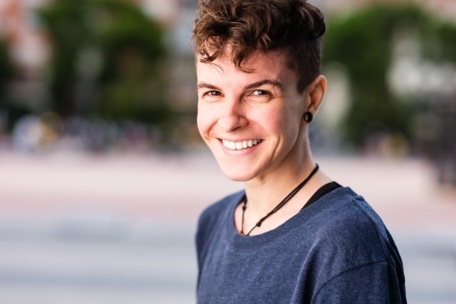 | 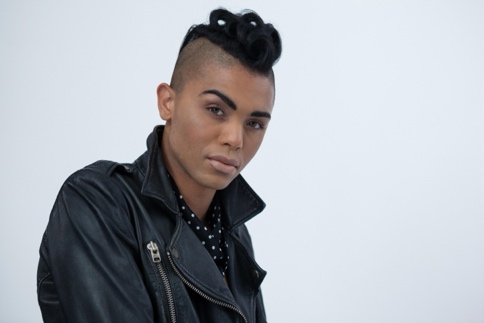 |
| 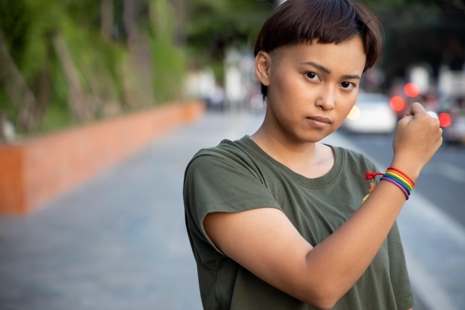 | 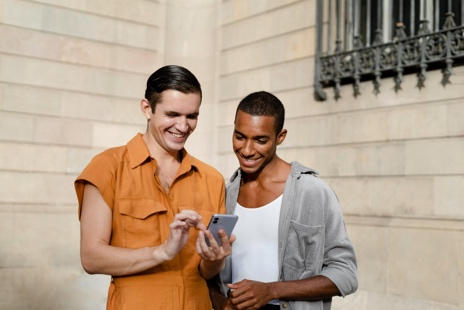 | 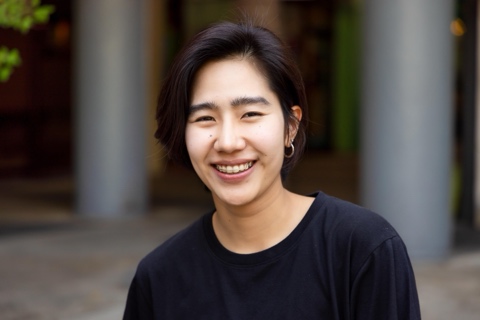 |
| 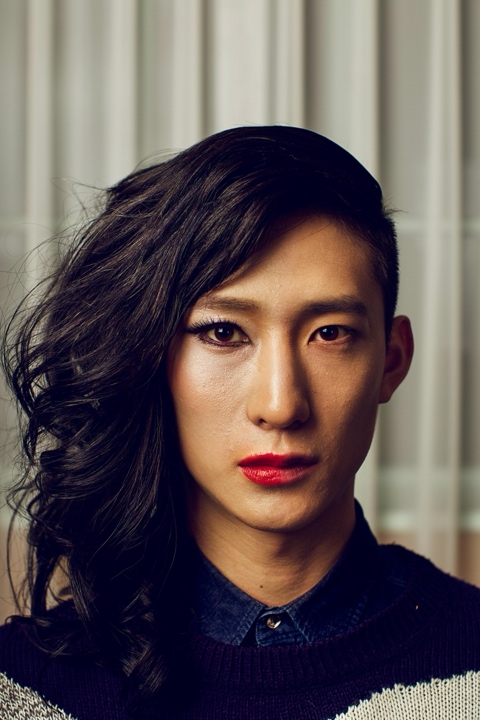 | 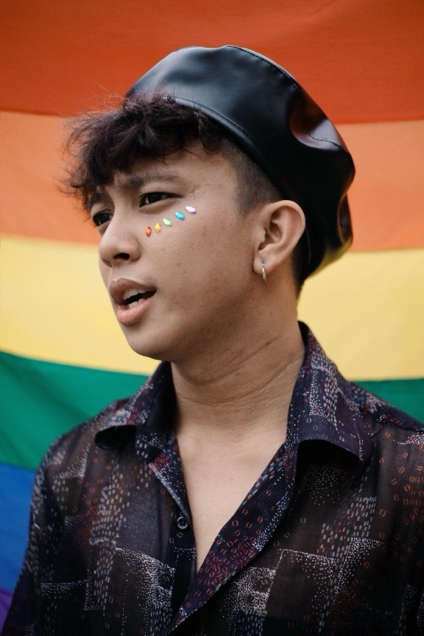 | 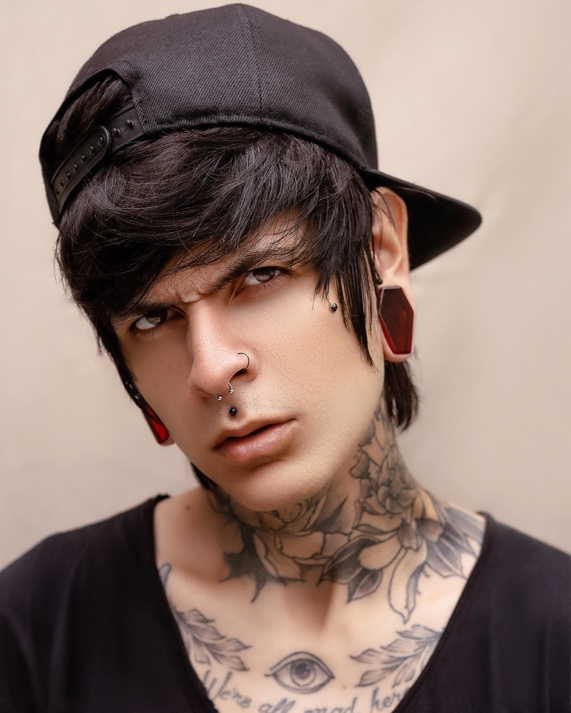 |

| Feminine expression | | |
| --- | --- | --- |
| 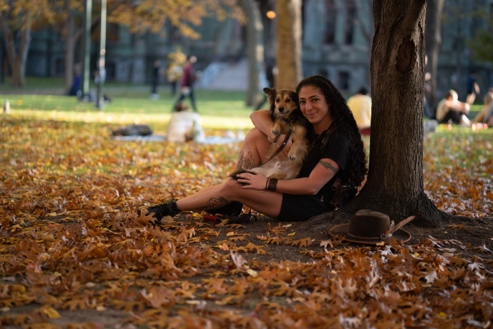 | 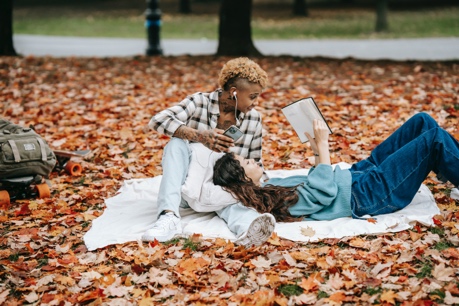 | 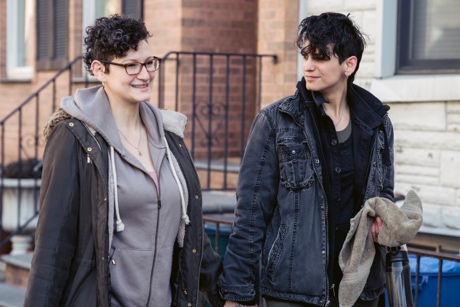 |
| 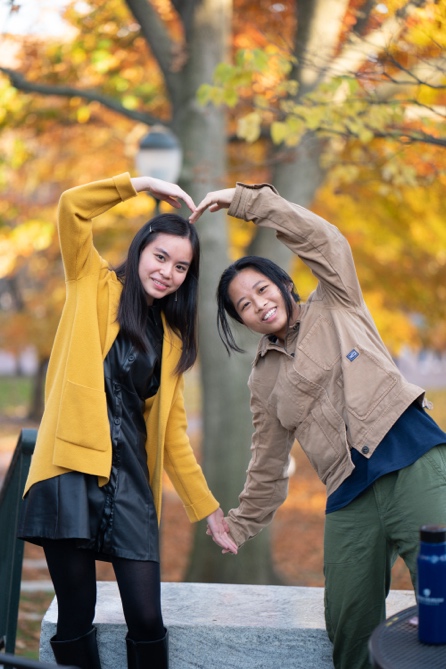 | 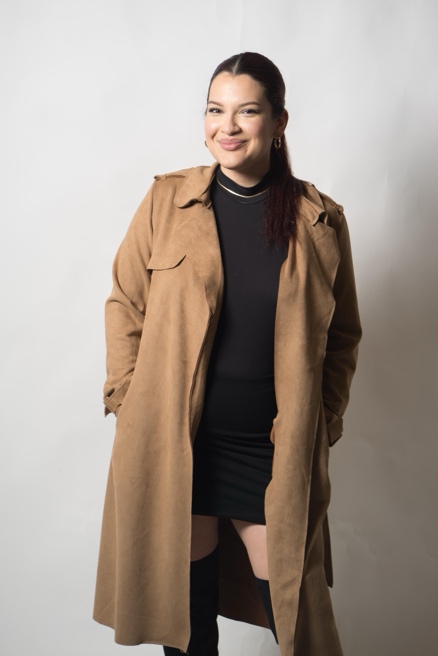 | 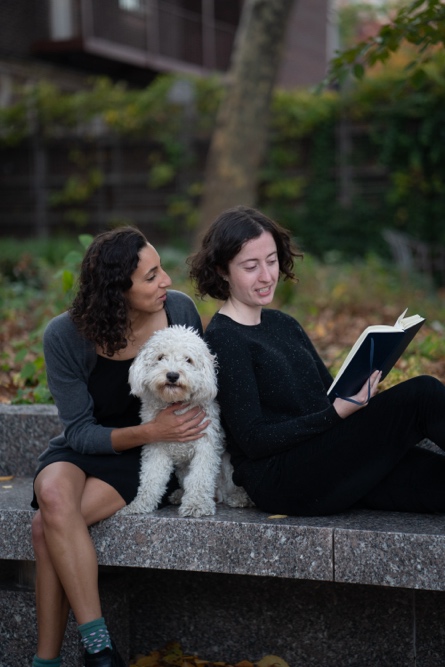 |
| 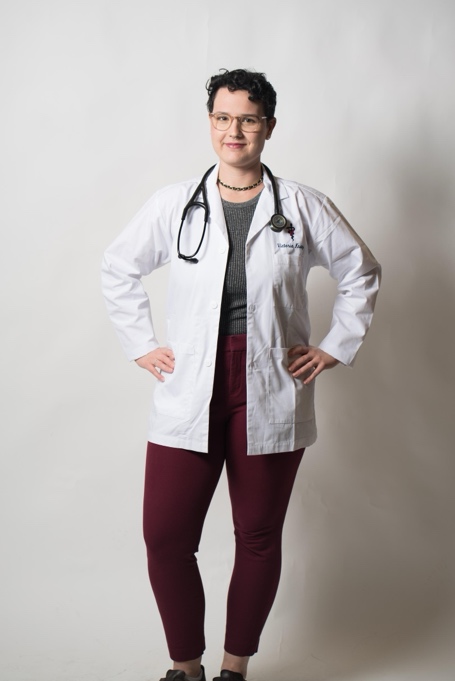 | 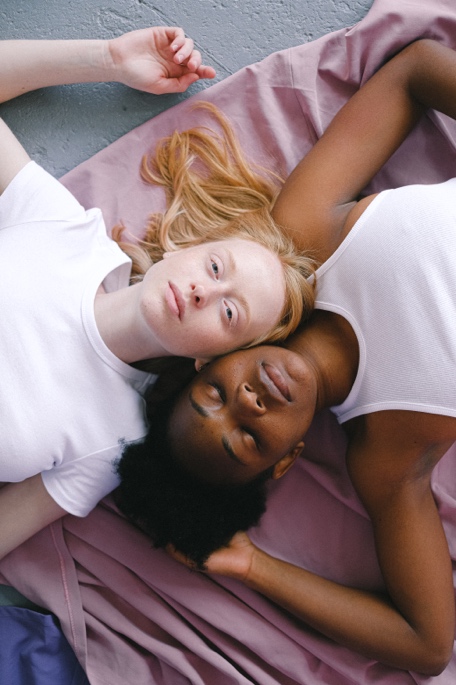 | 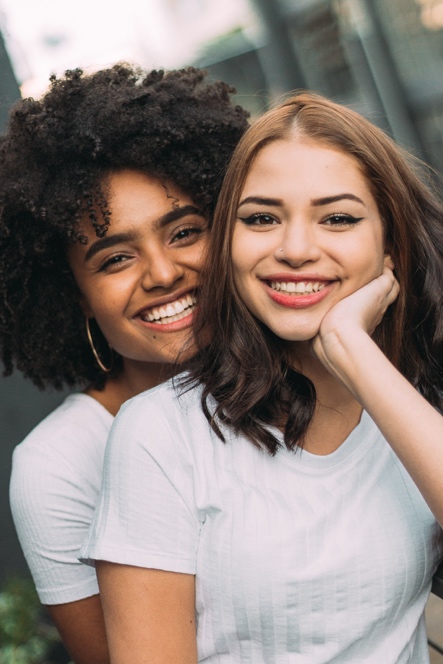 |

| Masculine expression | | |
| --- | --- | --- |
| 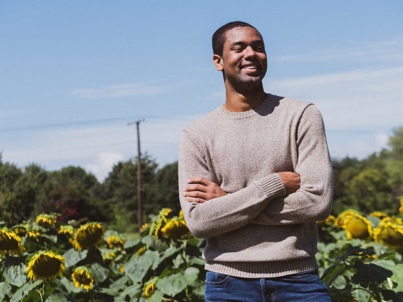 | 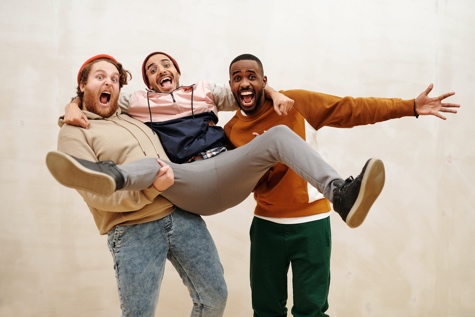 | 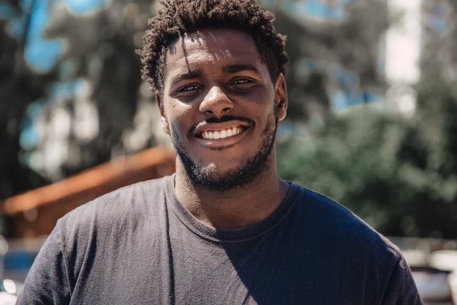 |
| 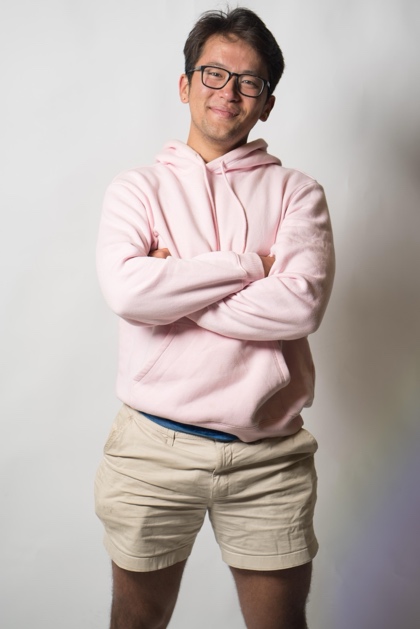 | 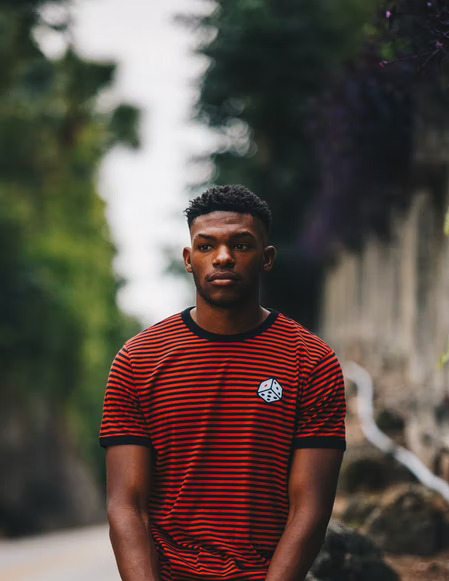 | 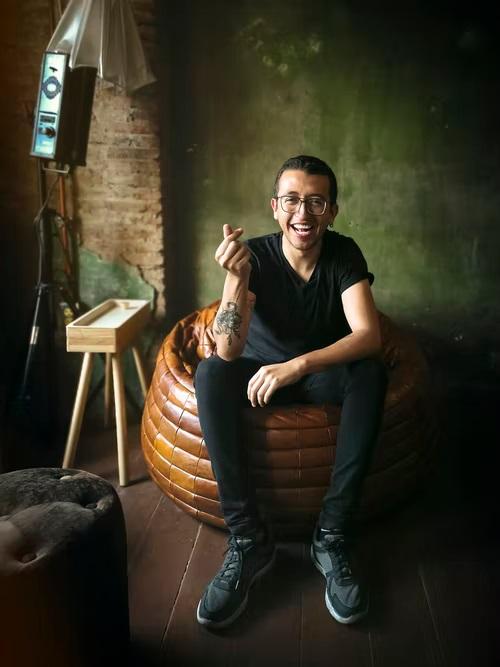 |
| 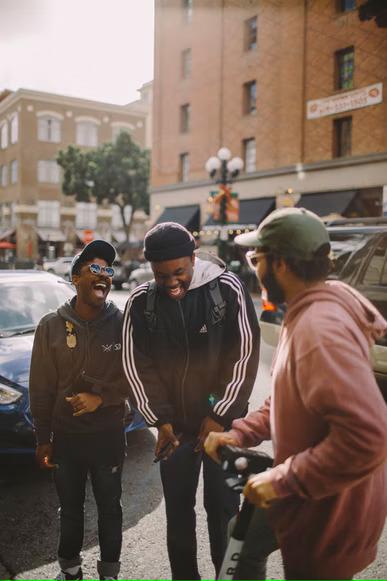 | 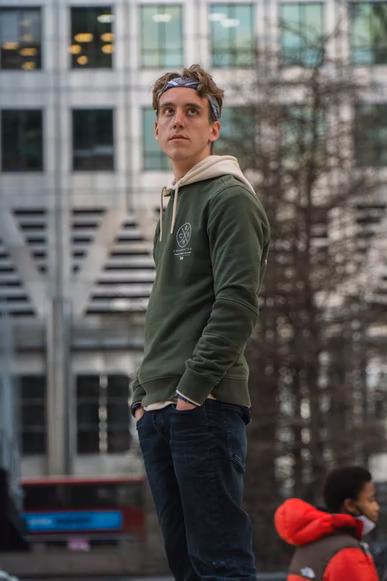 | 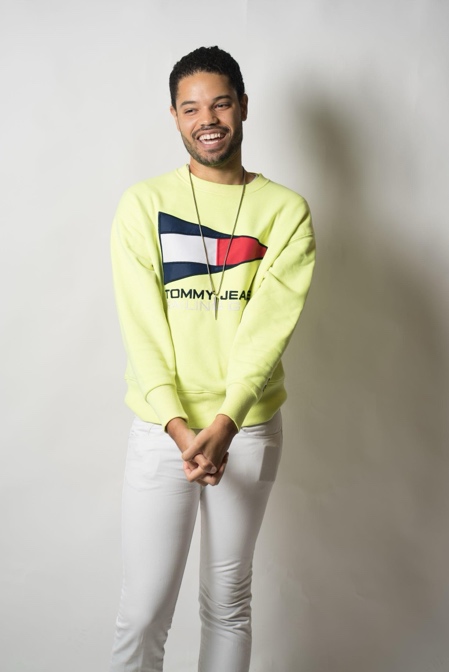 |

| Multiple gender expressions | | |
| --- | --- | --- |
| 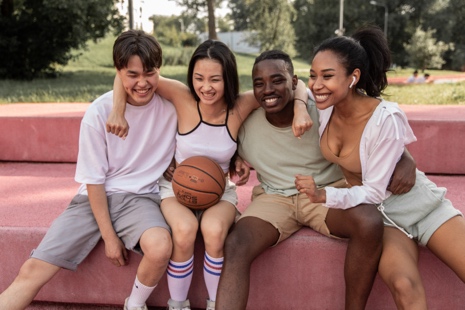 | 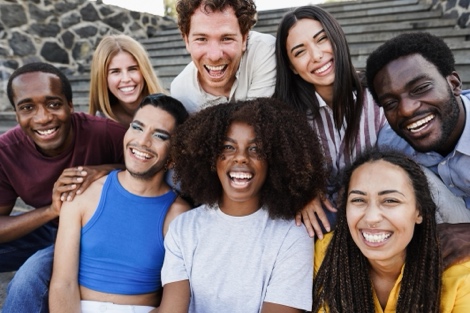 | 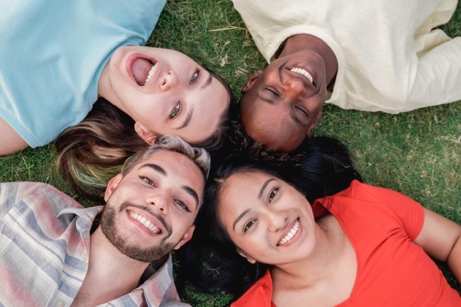 |
| 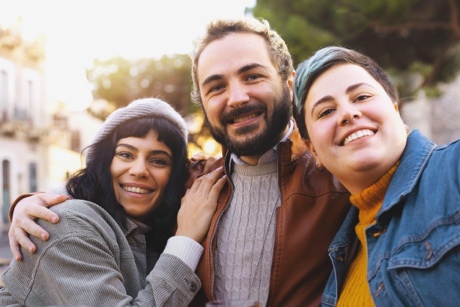 | 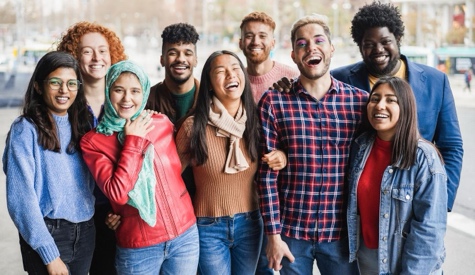 | 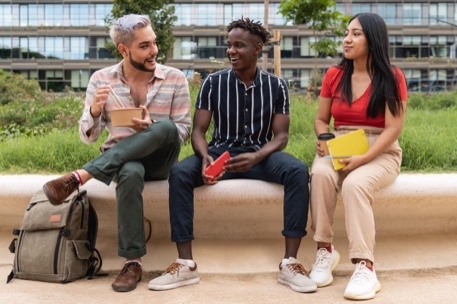 |
| 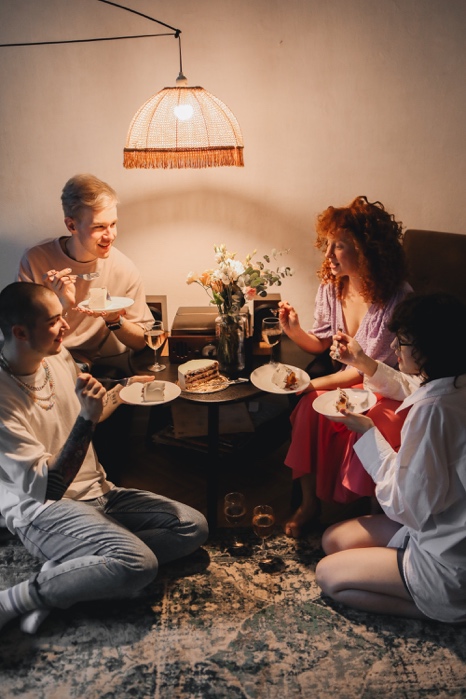 | 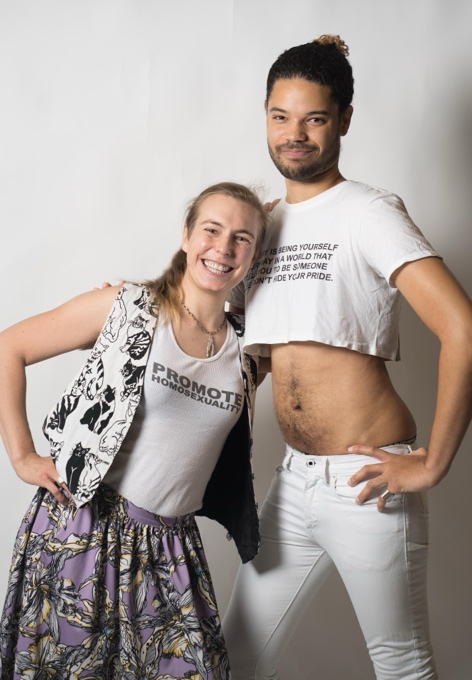 | 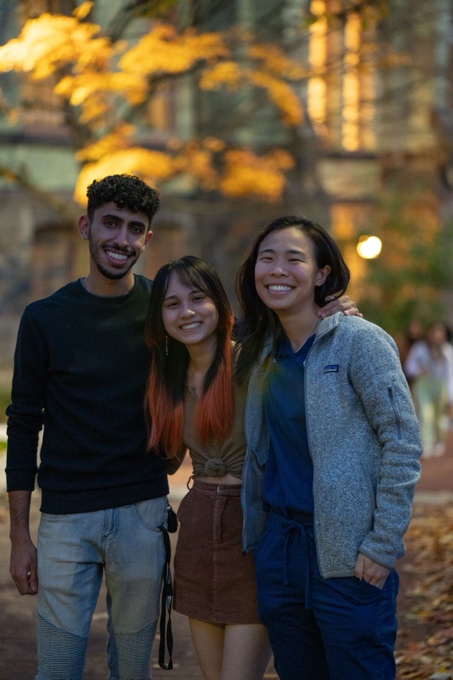 |

**Supplementary Materials: Study 2**

**Table S5**

Characteristics of Participants by Gender Identity (Study 2)

|  | Gender-expansive  (*N* = 327) | | | Trans feminine  (*N* = 25) | | | Trans masculine  (*N* = 55) | | | Cisgender SMW  (*N* = 389) | | | Cisgender SMM  (*N* = 394) | | |
| --- | --- | --- | --- | --- | --- | --- | --- | --- | --- | --- | --- | --- | --- | --- | --- |
|  | *M* (*SD*) | *n* | % | *M* (*SD*) | *n* | % | *M* (*SD*) | *n* | % | *M* (*SD*) | *n* | % | *M* (*SD*) | *n* | % |
| Age | 24.1 (3.4) |  |  | 24.6 (2.9) |  |  | 23.6 (3.0) |  |  | 24.7 (3.4) |  |  | 25.0 (3.2) |  |  |
| Sexual orientation |  |  |  |  |  |  |  |  |  |  |  |  |  |  |  |
| Gay or lesbian |  | 21 | 6.4 |  | 2 | 8.0 |  | 5 | 9.1 |  | 36 | 9.3 |  | 92 | 23.4 |
| Bisexual |  | 45 | 13.8 |  | 9 | 36.0 |  | 15 | 27.3 |  | 177 | 45.5 |  | 170 | 43.1 |
| Other^a^ |  | 261 | 79.8 |  | 14 | 56.0 |  | 35 | 63.6 |  | 176 | 45.2 |  | 132 | 33.5 |
| Race |  |  |  |  |  |  |  |  |  |  |  |  |  |  |  |
| White |  | 233 | 71.3 |  | 19 | 76.0 |  | 35 | 63.6 |  | 278 | 71.5 |  | 252 | 64.0 |
| Black |  | 13 | 4.0 |  | 0 | 0 |  | 2 | 3.6 |  | 41 | 10.5 |  | 49 | 12.4 |
| Other^b^ |  | 81 | 24.8 |  | 6 | 24.0 |  | 18 | 32.7 |  | 70 | 18.0 |  | 93 | 23.6 |
| Ethnicity |  |  |  |  |  |  |  |  |  |  |  |  |  |  |  |
| Hispanic |  | 45 | 13.8 |  | 4 | 16.0 |  | 10 | 18.2 |  | 52 | 13.4 |  | 59 | 15.0 |
| Non-Hispanic |  | 282 | 86.2 |  | 21 | 84.0 |  | 45 | 81.8 |  | 337 | 86.6 |  | 335 | 85.0 |
| Education |  |  |  |  |  |  |  |  |  |  |  |  |  |  |  |
| High school or less |  | 69 | 21.1 |  | 7 | 28.0 |  | 15 | 27.3 |  | 69 | 17.7 |  | 73 | 18.5 |
| Some college or associate degree |  | 149 | 45.6 |  | 13 | 72.0 |  | 26 | 47.3 |  | 151 | 38.8 |  | 148 | 37.6 |
| Bachelor’s degree |  | 86 | 26.3 |  | 5 | 20.0 |  | 14 | 25.5 |  | 144 | 37.0 |  | 137 | 34.8 |
| Graduate degree |  | 23 | 7.0 |  | 0 | 0 |  | 0 | 0 |  | 25 | 6.4 |  | 36 | 9.1 |
| Income |  |  |  |  |  |  |  |  |  |  |  |  |  |  |  |
| Less than $20, 000 |  | 81 | 24.8 |  | 10 | 40.0 |  | 8 | 14.5 |  | 70 | 18.0 |  | 61 | 15.5 |
| $ 20,000 to $ 49,999 |  | 109 | 33.3 |  | 8 | 32.0 |  | 21 | 38.2 |  | 116 | 29.8 |  | 113 | 28.7 |
| $ 50,000 to $ 74,999 |  | 55 | 16.8 |  | 1 | 4.0 |  | 7 | 12.7 |  | 95 | 24.4 |  | 80 | 20.3 |
| $ 75,000 to $ 99,999 |  | 35 | 10.7 |  | 1 | 4.0 |  | 5 | 9.1 |  | 49 | 12.6 |  | 58 | 14.7 |
| $ 100,000 or more |  | 47 | 14.4 |  | 5 | 20.0 |  | 14 | 25.5 |  | 58 | 14.9 |  | 82 | 20.8 |
| Smoker status |  |  |  |  |  |  |  |  |  |  |  |  |  |  |  |
| Current smoker |  | 57 | 17.4 |  | 3 | 12.0 |  | 12 | 21.8 |  | 61 | 15.7 |  | 82 | 20.8 |
| Not current smoker |  | 270 | 82.6 |  | 22 | 88.0 |  | 43 | 78.2 |  | 328 | 84.3 |  | 312 | 79.2 |

^a^Other includes 2 straight/heterosexual gender-expansive participants.

^b^Other includes Asian, American Indian or Alaska Native, Middle Eastern, Arab, or Arab American, Native Hawaiian, Other Pacific Islander, Samoan, and Mixed Race.

**Table S6**

Effects of Gender Expression on Transgender Image Perception (Study 2)

|  | *B* | *SE* |
| --- | --- | --- |
| Intercept | 0.90^***^ | 0.03 |
| Gender expression (Referent: Transgender/nonbinary expressions) |  |  |
| Feminine expressions | −0.88^***^ | 0.01 |
| Masculine expressions | −0.88^***^ | 0.01 |
| Multiple gender expressions | −0.88^***^ | 0.01 |
| Respondent gender identity (Referent: Gender-expansive/transgender identity) |  |  |
| Woman (cisgender SMW) | 0.00 | 0.01 |
| Man (cisgender SMM) | 0.01 | 0.01 |
| Age | −0.00 | 0.00 |
| Sexual orientation |  |  |
| Gay or lesbian | 0.02 | 0.01 |
| Other | 0.01 | 0.01 |
| Race |  |  |
| Black | −0.02 | 0.01 |
| Other | −0.01 | 0.01 |
| Ethnicity | 0.00 | 0.01 |
| Education |  |  |
| Some college | −0.00 | 0.01 |
| Bachelor’s degree | 0.01 | 0.01 |
| Graduate degree | −0.01 | 0.02 |
| Income | 0.00 | 0.00 |
| Smoker status | −0.01 | 0.01 |
| *F*(*df*) | 864.9^***^  (16, 1172) |  |
| *R*^2^ | .92 |  |
| adj. *R*^2^ | .92 |  |

*Note*. Transgender image perception is computed as the proportion of messages of being identified as including a transgender character in the condition. Results are obtained from the linear regression model conducted in R. Gender expression: Transgender/nonbinary expressions is reference category. Gender identity: Gender-expansive/transgender identity is reference category. Sexual orientation: Bisexual is reference category. Race: White is reference category. Ethnicity: Non-Hispanic is reference category. Education: High school or less is reference category. Smoker status: Not current smoker is reference category. Age and income are treated as continuous variables. ^+^ *p* < .08, ^*^ *p* < .05, ^**^ *p* < .01, ^***^ *p* < .001.

**Table S7.1**

Effects of Message Gender Expression and Respondent Gender Identity on Perceived Targetedness (Study 2)

|  | Main Effect | | Interaction Effect | |
| --- | --- | --- | --- | --- |
|  | *B* | *SE* | *B* | *SE* |
| Intercept | 2.10^***^ | 0.25 | 2.25^***^ | 0.26 |
| Gender expression (Referent: Transgender/nonbinary expressions) |  |  |  |  |
| Feminine expressions | 0.07 | 0.07 | −0.11 | 0.14 |
| Masculine expressions | −0.04 | 0.08 | −0.34^*^ | 0.15 |
| Multiple gender expressions | 0.13^+^ | 0.08 | −0.12 | 0.14 |
| Respondent gender identity (Referent: Gender-expansive identity) |  |  |  |  |
| Trans feminine | −0.05 | 0.19 | −0.14 | 0.38 |
| Trans masculine | −0.09 | 0.13 | −0.30 | 0.24 |
| Woman (cisgender SMW) | 0.04 | 0.07 | −0.13 | 0.14 |
| Man (cisgender SMM) | −0.13 | 0.07 | −0.47^***^ | 0.14 |
| LGBT image perception | 0.81^***^ | 0.09 | 0.82^***^ | 0.09 |
| Age | 0.01 | 0.01 | 0.01 | 0.01 |
| Sexual orientation |  |  |  |  |
| Gay or lesbian | 0.17^*^ | 0.09 | 0.17^*^ | 0.09 |
| Other | 0.07 | 0.06 | 0.07 | 0.06 |
| Race |  |  |  |  |
| Black | 0.17^+^ | 0.09 | 0.19^*^ | 0.09 |
| Other | −0.02 | 0.06 | −0.03 | 0.06 |
| Ethnicity | 0.03 | 0.08 | 0.04 | 0.08 |
| Education |  |  |  |  |
| Some college | 0.14^+^ | 0.07 | 0.13^+^ | 0.07 |
| Bachelor’s degree | 0.05 | 0.08 | 0.02 | 0.08 |
| Graduate degree | 0.08 | 0.12 | 0.07 | 0.12 |
| Income | −0.00 | 0.01 | 0.00 | 0.01 |
| Smoker status | 0.56^***^ | 0.07 | 0.56^***^ | 0.07 |
| Interaction |  |  |  |  |
| Trans feminine ×  Feminine expressions | ⎯ | ⎯ | 0.07 | 0.55 |
| Trans feminine ×  Masculine expressions | ⎯ | ⎯ | 0.14 | 0.51 |
| Trans feminine ×  Multiple gender expressions | ⎯ | ⎯ | 0.19 | 0.51 |
| Trans masculine ×  Feminine expressions | ⎯ | ⎯ | 0.13 | 0.38 |
| Trans masculine ×  Masculine expressions | ⎯ | ⎯ | 0.39 | 0.33 |
| Trans masculine ×  Multiple gender expressions | ⎯ | ⎯ | 0.29 | 0.41 |
| Woman (cisgender SMW) × Feminine expressions | ⎯ | ⎯ | 0.33^+^ | 0.19 |
| Woman (cisgender SMW) ×  Masculine expressions | ⎯ | ⎯ | 0.20 | 0.19 |
| Woman (cisgender SMW) × Multiple gender expressions | ⎯ | ⎯ | 0.13 | 0.19 |
| Man (cisgender SMM) ×  Feminine expressions | ⎯ | ⎯ | 0.18 | 0.19 |
| Man (cisgender SMM) ×  Masculine expressions | ⎯ | ⎯ | 0.61^**^ | 0.19 |
| Man (cisgender SMM) ×  Multiple gender expressions | ⎯ | ⎯ | 0.58^**^ | 0.19 |
| *F*(*df*) | 9.36^***^  (19, 1169) |  | 6.59^***^  (31, 1157) |  |
| *R*^2^ | .13 |  | .15 |  |
| adj. *R*^2^ | .12 |  | .13 |  |

*Note*. All coefficients are unstandardized. Gender expression: Transgender/nonbinary expressions is reference category. Gender identity: Gender-expansive identity is reference category. Sexual orientation: Bisexual is reference category. Race: White is reference category. Ethnicity: Non-Hispanic is reference category. Education: High school or less is reference category. Smoker status: Not current smoker is reference category. LGBT image perception, age and income are treated as continuous variables. ^+^ *p* < .08, ^*^ *p* < .05, ^**^ *p* < .01, ^***^ *p* < .001.

**Table S7.2**

Effects of Message Gender Expression and Respondent Gender Identity on Perceived Targetedness (Study 2)

|  | Main Effect | | Interaction Effect | |
| --- | --- | --- | --- | --- |
|  | *B* | *SE* | *B* | *SE* |
| Intercept | 2.05^***^ | 0.31 | 2.10^***^ | 0.43 |
| Gender expression (Referent: Transgender/nonbinary expressions) |  |  |  |  |
| Feminine expressions | 0.07 | 0.07 | −0.04 | 0.54 |
| Masculine expressions | −0.04 | 0.08 | −0.20 | 0.49 |
| Multiple gender expressions | 0.13^+^ | 0.08 | 0.07 | 0.50 |
| Respondent gender identity (Referent: Trans feminine identity) |  |  |  |  |
| Gender-expansive | 0.05 | 0.19 | 0.14 | 0.38 |
| Trans masculine | −0.04 | 0.22 | −0.16 | 0.42 |
| Woman (cisgender SMW) | 0.08 | 0.18 | 0.01 | 0.37 |
| Man (cisgender SMM) | −0.08 | 0.19 | −0.33 | 0.37 |
| LGBT image perception | 0.81^***^ | 0.09 | 0.82^***^ | 0.09 |
| Age | 0.01 | 0.01 | 0.01 | 0.01 |
| Sexual orientation |  |  |  |  |
| Gay or lesbian | 0.17^*^ | 0.09 | 0.17^*^ | 0.09 |
| Other | 0.07 | 0.06 | 0.07 | 0.06 |
| Race |  |  |  |  |
| Black | 0.17^+^ | 0.09 | 0.19^*^ | 0.09 |
| Other | −0.02 | 0.06 | −0.03 | 0.06 |
| Ethnicity | 0.03 | 0.08 | 0.04 | 0.08 |
| Education |  |  |  |  |
| Some college | 0.14^+^ | 0.07 | 0.13^+^ | 0.07 |
| Bachelor’s degree | 0.05 | 0.08 | 0.02 | 0.08 |
| Graduate degree | 0.08 | 0.12 | 0.07 | 0.12 |
| Income | −0.00 | 0.01 | 0.00 | 0.01 |
| Smoker status | 0.56^***^ | 0.07 | 0.56^***^ | 0.07 |
| Interaction |  |  |  |  |
| Gender-expansive × Feminine expressions | ⎯ | ⎯ | −0.07 | 0.55 |
| Gender-expansive × Masculine expressions | ⎯ | ⎯ | −0.14 | 0.51 |
| Gender-expansive × Multiple gender expressions | ⎯ | ⎯ | −0.19 | 0.51 |
| Trans masculine × Feminine expressions | ⎯ | ⎯ | 0.06 | 0.64 |
| Trans masculine × Masculine expressions | ⎯ | ⎯ | 0.25 | 0.58 |
| Trans masculine × Multiple gender expressions | ⎯ | ⎯ | 0.10 | 0.63 |
| Woman (cisgender SMW) × Feminine expressions | ⎯ | ⎯ | 0.26 | 0.55 |
| Woman (cisgender SMW) × Masculine expressions | ⎯ | ⎯ | 0.06 | 0.51 |
| Woman (cisgender SMW) × Multiple gender expressions | ⎯ | ⎯ | −0.06 | 0.51 |
| Man (cisgender SMM) × Feminine expressions | ⎯ | ⎯ | 0.11 | 0.55 |
| Man (cisgender SMM) × Masculine expressions | ⎯ | ⎯ | 0.48 | 0.51 |
| Man (cisgender SMM) × Multiple gender expressions | ⎯ | ⎯ | 0.40 | 0.51 |
| *F*(*df*) | 9.36^***^  (19, 1169) |  | 6.59^***^  (31, 1157) |  |
| *R*^2^ | .13 |  | .15 |  |
| adj. *R*^2^ | .12 |  | .13 |  |

*Note*. All coefficients are unstandardized. Gender expression: Transgender/nonbinary expressions is reference category. Gender identity: Trans feminine identity is reference category. Sexual orientation: Bisexual is reference category. Race: White is reference category. Ethnicity: Non-Hispanic is reference category. Education: High school or less is reference category. Smoker status: Not current smoker is reference category. LGBT image perception, age and income are treated as continuous variables. ^+^ *p* < .08, ^*^ *p* < .05, ^**^ *p* < .01, ^***^ *p* < .001.

**Table S7.3**

Effects of Message Gender Expression and Respondent Gender Identity on Perceived Targetedness (Study 2)

|  | Main Effect | | Interaction Effect | |
| --- | --- | --- | --- | --- |
|  | *B* | *SE* | *B* | *SE* |
| Intercept | 2.01^***^ | 0.27 | 1.95^***^ | 0.33 |
| Gender expression (Referent: Transgender/nonbinary expressions) |  |  |  |  |
| Feminine expressions | 0.07 | 0.07 | 0.02 | 0.36 |
| Masculine expressions | −0.04 | 0.08 | 0.05 | 0.30 |
| Multiple gender expressions | 0.13^+^ | 0.08 | 0.17 | 0.39 |
| Respondent gender identity (Referent: Trans masculine identity) |  |  |  |  |
| Gender-expansive | 0.09 | 0.13 | 0.30 | 0.24 |
| Trans feminine | 0.04 | 0.22 | 0.16 | 0.42 |
| Woman (cisgender SMW) | 0.12 | 0.13 | 0.17 | 0.24 |
| Man (cisgender SMM) | −0.04 | 0.13 | −0.17 | 0.24 |
| LGBT image perception | 0.81^***^ | 0.09 | 0.82^***^ | 0.09 |
| Age | 0.01 | 0.01 | 0.01 | 0.01 |
| Sexual orientation |  |  |  |  |
| Gay or lesbian | 0.17^*^ | 0.09 | 0.17^*^ | 0.09 |
| Other | 0.07 | 0.06 | 0.07 | 0.06 |
| Race |  |  |  |  |
| Black | 0.17^+^ | 0.09 | 0.19^*^ | 0.09 |
| Other | −0.02 | 0.06 | −0.03 | 0.06 |
| Ethnicity | 0.03 | 0.08 | 0.04 | 0.08 |
| Education |  |  |  |  |
| Some college | 0.14^+^ | 0.07 | 0.13^+^ | 0.07 |
| Bachelor’s degree | 0.05 | 0.08 | 0.02 | 0.08 |
| Graduate degree | 0.08 | 0.12 | 0.07 | 0.12 |
| Income | −0.00 | 0.01 | 0.00 | 0.01 |
| Smoker status | 0.56^***^ | 0.07 | 0.56^***^ | 0.07 |
| Interaction |  |  |  |  |
| Gender-expansive × Feminine expressions | ⎯ | ⎯ | −0.13 | 0.38 |
| Gender-expansive × Masculine expressions | ⎯ | ⎯ | −0.39 | 0.33 |
| Gender-expansive × Multiple gender expressions | ⎯ | ⎯ | −0.29 | 0.41 |
| Trans feminine × Feminine expressions | ⎯ | ⎯ | −0.06 | 0.64 |
| Trans feminine × Masculine expressions | ⎯ | ⎯ | −0.25 | 0.58 |
| Trans feminine × Multiple gender expressions | ⎯ | ⎯ | −0.10 | 0.63 |
| Woman (cisgender SMW) × Feminine expressions | ⎯ | ⎯ | 0.20 | 0.38 |
| Woman (cisgender SMW) × Masculine expressions | ⎯ | ⎯ | −0.19 | 0.32 |
| Woman (cisgender SMW) × Multiple gender expressions | ⎯ | ⎯ | −0.15 | 0.41 |
| Man (cisgender SMM) × Feminine expressions | ⎯ | ⎯ | 0.05 | 0.38 |
| Man (cisgender SMM) × Masculine expressions | ⎯ | ⎯ | 0.23 | 0.32 |
| Man (cisgender SMM) × Multiple gender expressions | ⎯ | ⎯ | 0.30 | 0.40 |
| *F*(*df*) | 9.36^***^  (19, 1169) |  | 6.59^***^  (31, 1157) |  |
| *R*^2^ | .13 |  | .15 |  |
| adj. *R*^2^ | .12 |  | .13 |  |

*Note*. All coefficients are unstandardized. Gender expression: Transgender/nonbinary expressions is reference category. Gender identity: Trans masculine identity is reference category. Sexual orientation: Bisexual is reference category. Race: White is reference category. Ethnicity: Non-Hispanic is reference category. Education: High school or less is reference category. Smoker status: Not current smoker is reference category. LGBT image perception, age and income are treated as continuous variables. ^+^ *p* < .08, ^*^ *p* < .05, ^**^ *p* < .01, ^***^ *p* < .001.

**Table S7.4**

Effects of Message Gender Expression and Respondent Gender Identity on Perceived Targetedness (Study 2)

|  | Main Effect | | Interaction Effect | |
| --- | --- | --- | --- | --- |
|  | *B* | *SE* | *B* | *SE* |
| Intercept | 2.20^***^ | 0.25 | 2.34^***^ | 0.25 |
| Gender expression (Referent: Feminine expressions) |  |  |  |  |
| Transgender/nonbinary expressions | −0.07 | 0.07 | −0.22^+^ | 0.13 |
| Masculine expressions | −0.11 | 0.07 | −0.36^**^ | 0.13 |
| Multiple gender expressions | 0.07 | 0.07 | −0.21 | 0.13 |
| Respondent gender identity (Referent: Woman (cisgender SMW)) |  |  |  |  |
| Gender-expansive | −0.04 | 0.07 | −0.20 | 0.13 |
| Trans feminine | −0.08 | 0.18 | −0.28 | 0.41 |
| Trans masculine | −0.12 | 0.13 | −0.37 | 0.30 |
| Man (cisgender SMM) | −0.16 | 0.06 | −0.50^***^ | 0.13 |
| LGBT image perception | 0.81^***^ | 0.09 | 0.82^***^ | 0.09 |
| Age | 0.01 | 0.01 | 0.01 | 0.01 |
| Sexual orientation |  |  |  |  |
| Gay or lesbian | 0.17^*^ | 0.09 | 0.17^*^ | 0.09 |
| Other | 0.07 | 0.06 | 0.07 | 0.06 |
| Race |  |  |  |  |
| Black | 0.17^+^ | 0.09 | 0.19^*^ | 0.09 |
| Other | −0.02 | 0.06 | −0.03 | 0.06 |
| Ethnicity | 0.03 | 0.08 | 0.04 | 0.08 |
| Education |  |  |  |  |
| Some college | 0.14^+^ | 0.07 | 0.13^+^ | 0.07 |
| Bachelor’s degree | 0.05 | 0.08 | 0.02 | 0.08 |
| Graduate degree | 0.08 | 0.12 | 0.07 | 0.12 |
| Income | −0.00 | 0.01 | 0.00 | 0.01 |
| Smoker status | 0.56^***^ | 0.07 | 0.56^***^ | 0.07 |
| Interaction |  |  |  |  |
| Gender-expansive × Transgender/nonbinary expressions | ⎯ | ⎯ | 0.33^+^ | 0.19 |
| Gender-expansive × Masculine expressions | ⎯ | ⎯ | 0.13 | 0.19 |
| Gender-expansive × Multiple gender expressions | ⎯ | ⎯ | 0.20 | 0.19 |
| Trans feminine × Transgender/nonbinary expressions | ⎯ | ⎯ | 0.26 | 0.55 |
| Trans feminine × Masculine expressions | ⎯ | ⎯ | 0.20 | 0.53 |
| Trans feminine × Multiple gender expressions | ⎯ | ⎯ | 0.32 | 0.54 |
| Trans masculine × Transgender/nonbinary expressions | ⎯ | ⎯ | 0.20 | 0.38 |
| Trans masculine × Masculine expressions | ⎯ | ⎯ | 0.39 | 0.36 |
| Trans masculine × Multiple gender expressions | ⎯ | ⎯ | 0.36 | 0.44 |
| Man (cisgender SMM) × Transgender/nonbinary expressions | ⎯ | ⎯ | 0.16 | 0.18 |
| Man (cisgender SMM) × Masculine expressions | ⎯ | ⎯ | 0.57^**^ | 0.18 |
| Man (cisgender SMM) × Multiple gender expressions | ⎯ | ⎯ | 0.61^***^ | 0.18 |
| *F*(*df*) | 9.36^***^  (19, 1169) |  | 6.59^***^  (31, 1157) |  |
| *R*^2^ | .13 |  | .15 |  |
| adj. *R*^2^ | .12 |  | .13 |  |

*Note*. All coefficients are unstandardized. Gender expression: Feminine expressions is reference category. Gender identity: Woman (cisgender SMW) identity is reference category. Sexual orientation: Bisexual is reference category. Race: White is reference category. Ethnicity: Non-Hispanic is reference category. Education: High school or less is reference category. Smoker status: Not current smoker is reference category. LGBT image perception, age and income are treated as continuous variables. ^+^ *p* < .08, ^*^ *p* < .05, ^**^ *p* < .01, ^***^ *p* < .001.

**Table S7.5**

Effects of Message Gender Expression and Respondent Gender Identity on Perceived Targetedness (Study 2)

|  | Main Effect | | Interaction Effect | |
| --- | --- | --- | --- | --- |
|  | *B* | *SE* | *B* | *SE* |
| Intercept | 1.93^***^ | 0.25 | 2.06^***^ | 0.25 |
| Gender expression (Referent: Masculine expressions) |  |  |  |  |
| Transgender/nonbinary expressions | 0.04 | 0.08 | −.28^*^ | 0.13 |
| Feminine expressions | 0.11 | 0.07 | −.21 | 0.13 |
| Multiple gender expressions | 0.17^*^ | 0.07 | .19 | 0.13 |
| Respondent gender identity (Referent: Man (cisgender SMM)) |  |  |  |  |
| Gender-expansive | 0.13 | 0.07 | −.14 | 0.14 |
| Trans feminine | 0.08 | 0.19 | −.15 | 0.35 |
| Trans masculine | 0.04 | 0.13 | −.06 | 0.22 |
| Woman (cisgender SMW) | 0.16 | 0.06 | −.08 | 0.13 |
| LGBT image perception | 0.81^***^ | 0.09 | 0.82^***^ | 0.09 |
| Age | 0.01 | 0.01 | 0.01 | 0.01 |
| Sexual orientation |  |  |  |  |
| Gay or lesbian | 0.17^*^ | 0.09 | 0.17^*^ | 0.09 |
| Other | 0.07 | 0.06 | 0.07 | 0.06 |
| Race |  |  |  |  |
| Black | 0.17^+^ | 0.09 | 0.19^*^ | 0.09 |
| Other | −0.02 | 0.06 | −0.03 | 0.06 |
| Ethnicity | 0.03 | 0.08 | 0.04 | 0.08 |
| Education |  |  |  |  |
| Some college | 0.14^+^ | 0.07 | 0.13^+^ | 0.07 |
| Bachelor’s degree | 0.05 | 0.08 | 0.02 | 0.08 |
| Graduate degree | 0.08 | 0.12 | 0.07 | 0.12 |
| Income | −0.00 | 0.01 | 0.00 | 0.01 |
| Smoker status | 0.56^***^ | 0.07 | 0.56^***^ | 0.07 |
| Interaction |  |  |  |  |
| Gender-expansive × Transgender/nonbinary expressions | ⎯ | ⎯ | 0.61^**^ | 0.19 |
| Gender-expansive × Feminine expressions | ⎯ | ⎯ | 0.44^*^ | 0.19 |
| Gender-expansive × Multiple gender expressions | ⎯ | ⎯ | 0.03 | 0.19 |
| Trans feminine × Transgender/nonbinary expressions | ⎯ | ⎯ | 0.48 | 0.51 |
| Trans feminine × Feminine expressions | ⎯ | ⎯ | 0.37 | 0.53 |
| Trans feminine × Multiple gender expressions | ⎯ | ⎯ | 0.08 | 0.49 |
| Trans masculine × Transgender/nonbinary expressions | ⎯ | ⎯ | 0.23 | 0.32 |
| Trans masculine × Feminine expressions | ⎯ | ⎯ | 0.18 | 0.36 |
| Trans masculine × Multiple gender expressions | ⎯ | ⎯ | −0.07 | 0.39 |
| Woman (cisgender SMW) × Transgender/nonbinary expressions | ⎯ | ⎯ | 0.42^*^ | 0.18 |
| Woman (cisgender SMW) × Feminine expressions | ⎯ | ⎯ | 0.57^**^ | 0.18 |
| Woman (cisgender SMW) × Multiple gender expressions | ⎯ | ⎯ | −0.04 | 0.18 |
| *F*(*df*) | 9.36^***^  (19, 1169) |  | 6.59^***^  (31, 1157) |  |
| *R*^2^ | .13 |  | .15 |  |
| adj. *R*^2^ | .12 |  | .13 |  |

*Note*. All coefficients are unstandardized. Gender expression: Masculine expressions is reference category. Gender identity: Man (cisgender SMM) identity is reference category. Sexual orientation: Bisexual is reference category. Race: White is reference category. Ethnicity: Non-Hispanic is reference category. Education: High school or less is reference category. Smoker status: Not current smoker is reference category. LGBT image perception, age and income are treated as continuous variables. ^+^ *p* < .08, ^*^ *p* < .05, ^**^ *p* < .01, ^***^ *p* < .001.

**Table S8**

Marginal Means of Perceived Targetedness (Study 2)

| Gender expression | Gender-expansive | | | Trans feminine | | | Trans masculine | | | Cisgender SMW | | | Cisgender SMM | | |
| --- | --- | --- | --- | --- | --- | --- | --- | --- | --- | --- | --- | --- | --- | --- | --- |
|  | *M* | 95% *CI* | | *M* | 95% *CI* | | *M* | 95% *CI* | | *M* | 95% *CI* | | *M* | 95% *CI* | |
|  |  | *LL* | *UL* |  | *LL* | *UL* |  | *LL* | *UL* |  | *LL* | *UL* |  | *LL* | *UL* |
| Transgender/nonbinary expressions | 3.32 | 3.12 | 3.52 | 3.18 | 2.47 | 3.89 | 3.02 | 2.58 | 3.46 | 3.19 | 3.01 | 3.37 | 2.85 | 2.67 | 3.03 |
| Feminine expressions | 3.22 | 3.03 | 3.40 | 3.14 | 2.36 | 3.92 | 3.04 | 2.49 | 3.59 | 3.42 | 3.24 | 3.59 | 2.92 | 2.75 | 3.09 |
| Masculine expressions | 2.99 | 2.78 | 3.20 | 2.98 | 2.32 | 3.64 | 3.07 | 2.69 | 3.46 | 3.05 | 2.87 | 3.23 | 3.13 | 2.95 | 3.31 |
| Multiple gender expressions | 3.20 | 3.01 | 3.39 | 3.25 | 2.58 | 3.91 | 3.19 | 2.57 | 3.80 | 3.20 | 3.03 | 3.38 | 3.32 | 3.14 | 3.50 |

**Figure S2**

Messages Representing Each Gender Expression (Study 2)

| Transgender/nonbinary expression | | |
| --- | --- | --- |
| *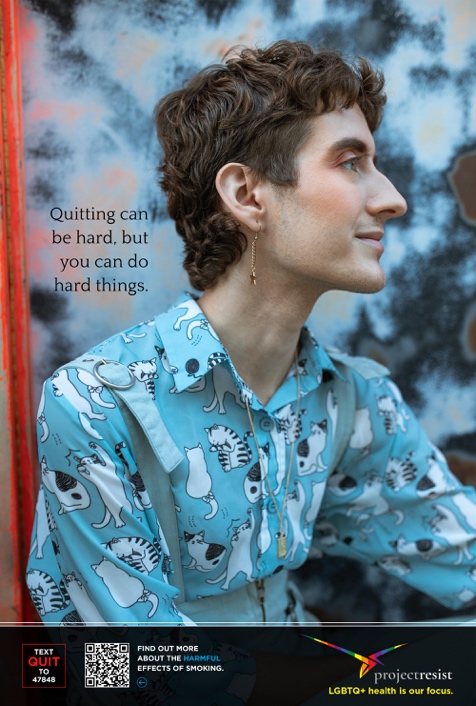* | 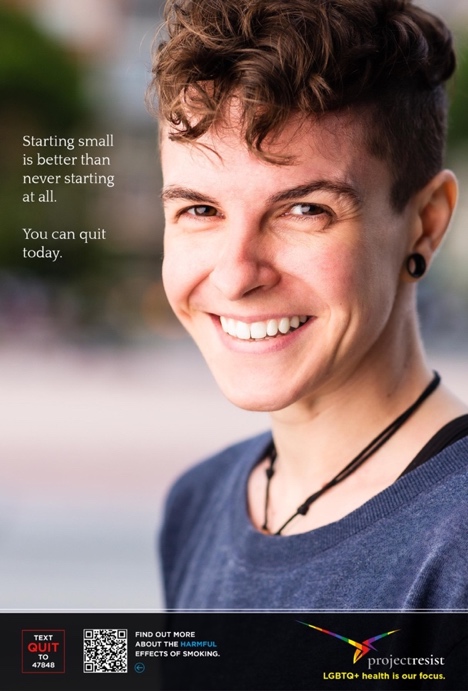 | *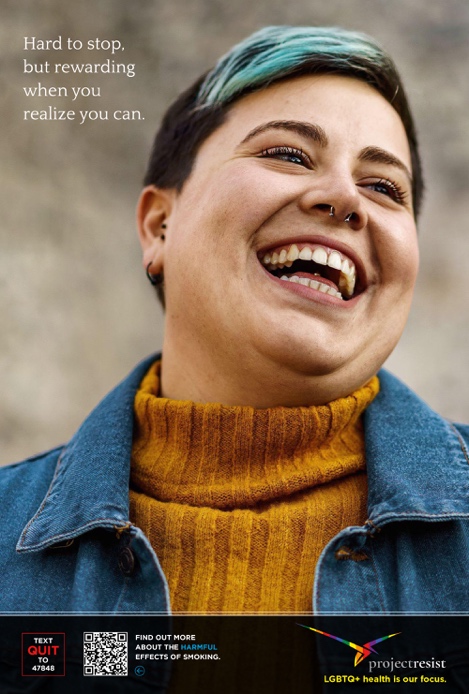* |
| *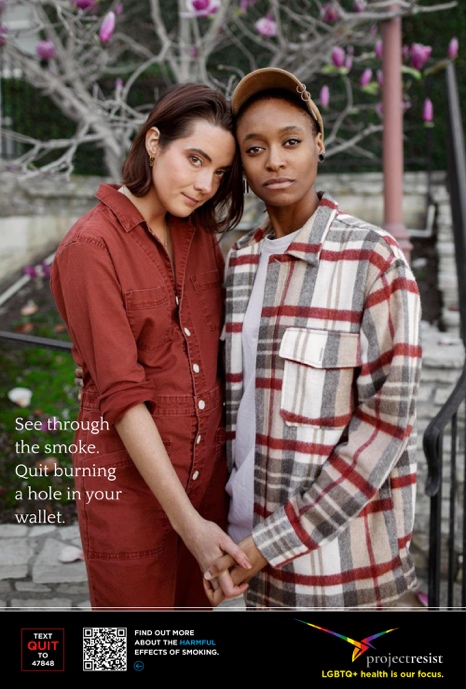* | *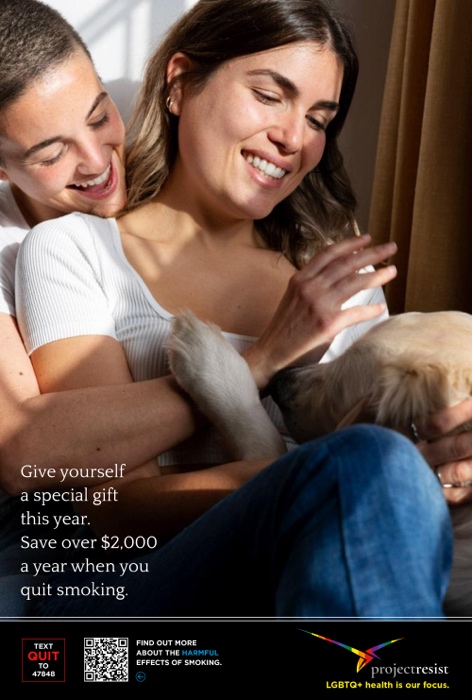* | *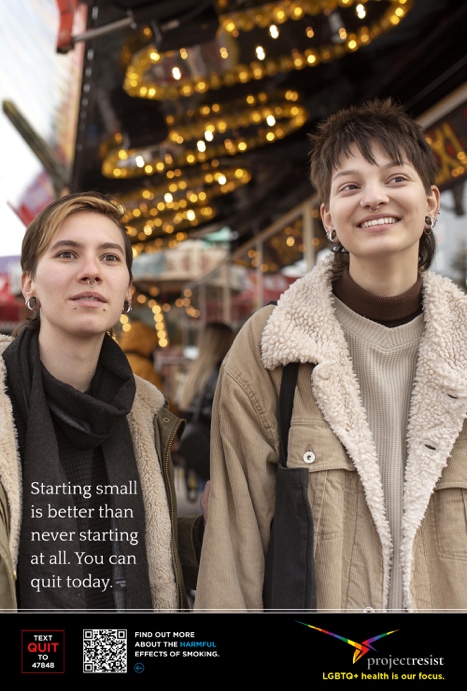* |

| Feminine expression | | |
| --- | --- | --- |
| *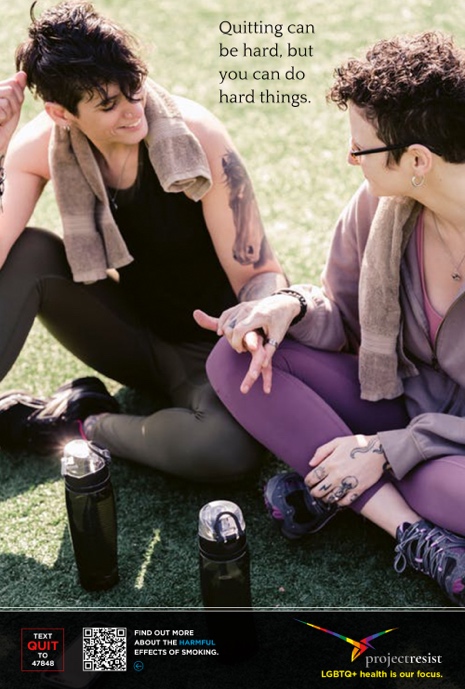* | *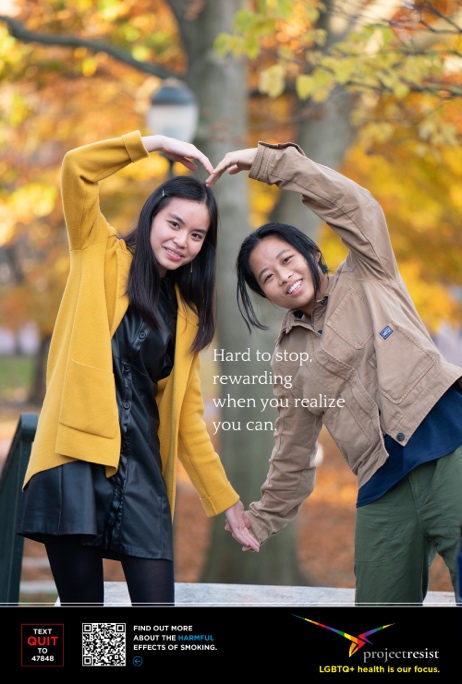* | *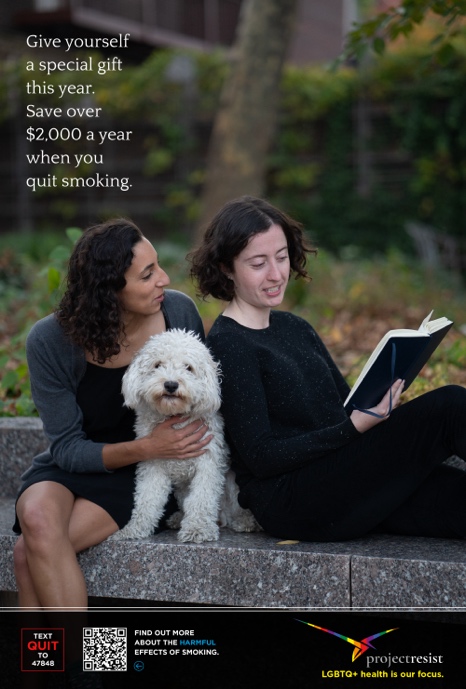* |
| *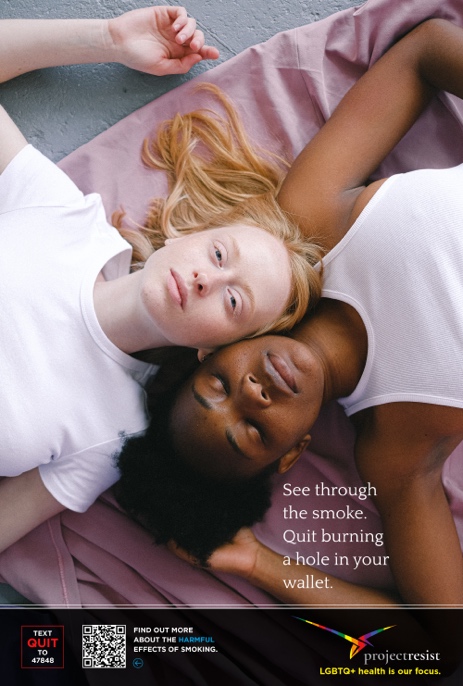* | *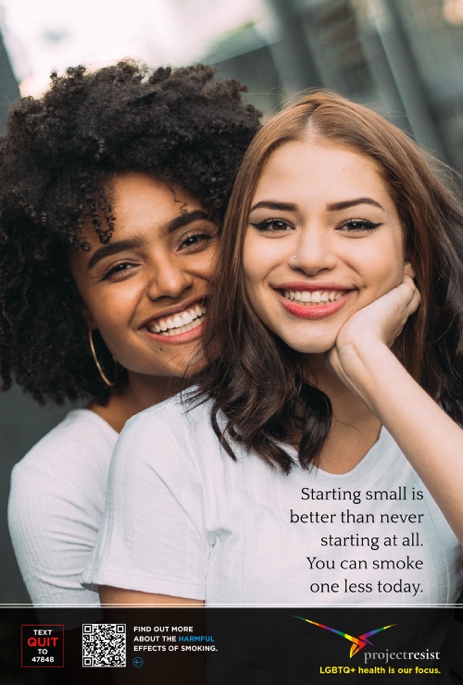* | *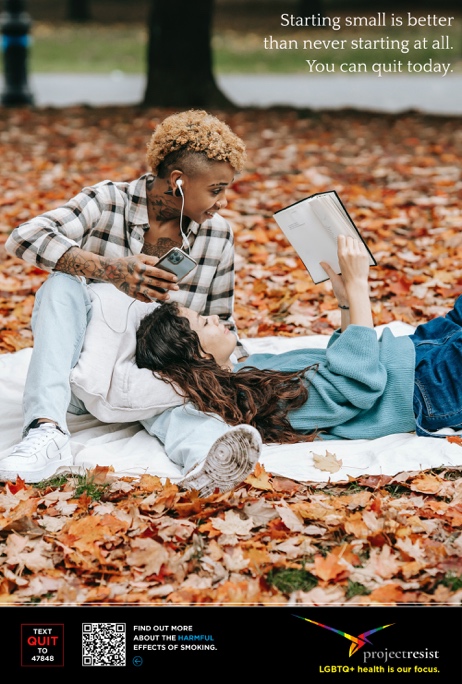* |

| Masculine expression | | |
| --- | --- | --- |
| *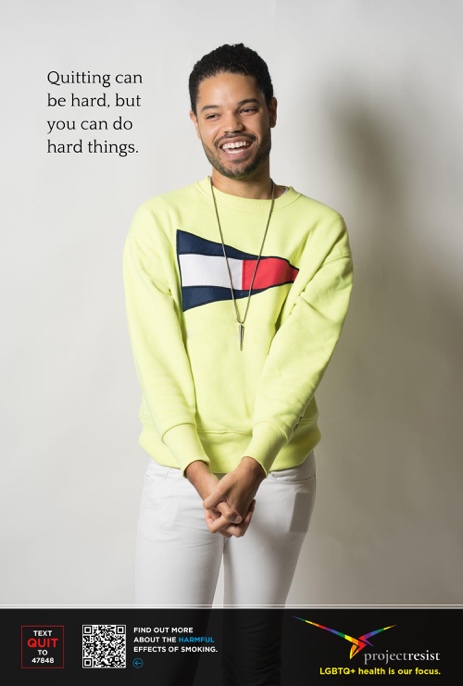* | *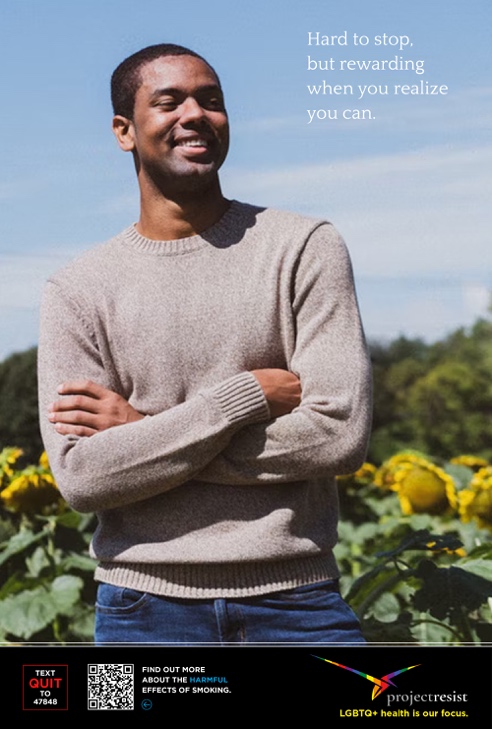* | *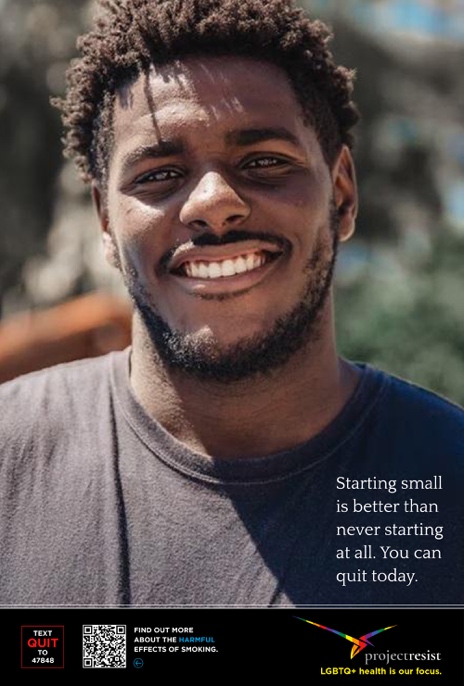* |
| *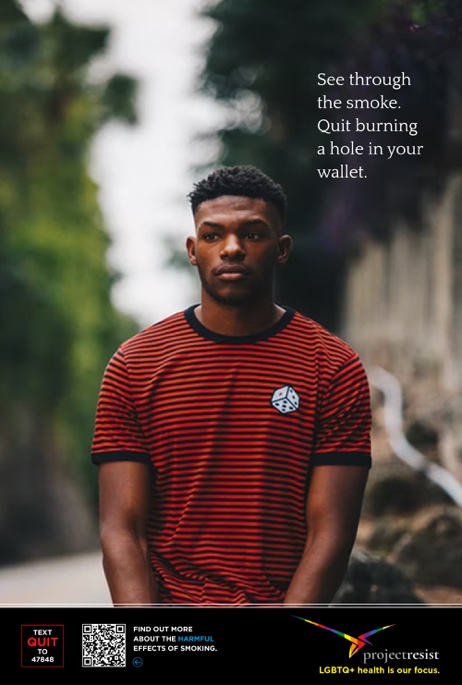* | *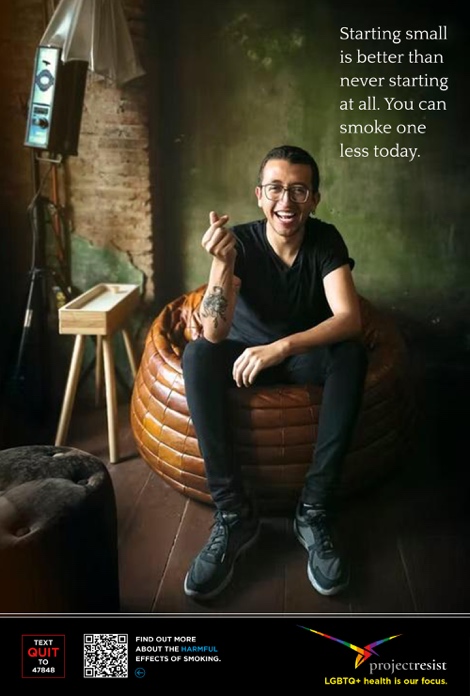* | *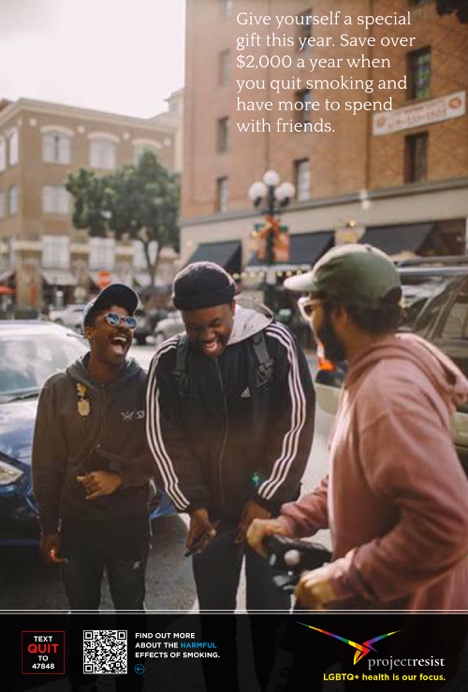* |

| Multiple gender expressions | | |
| --- | --- | --- |
| *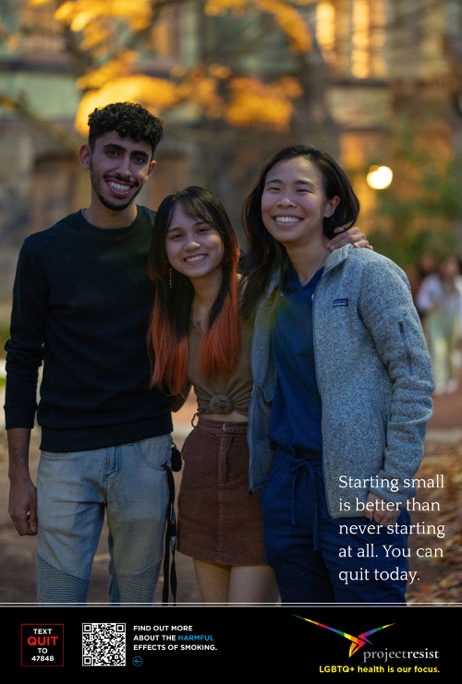* | *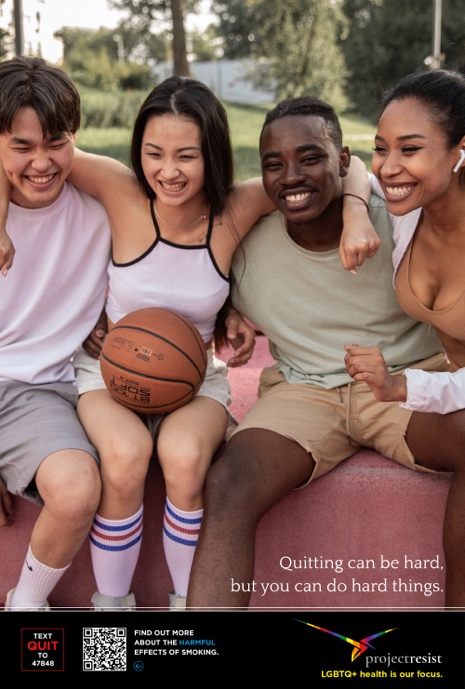* | *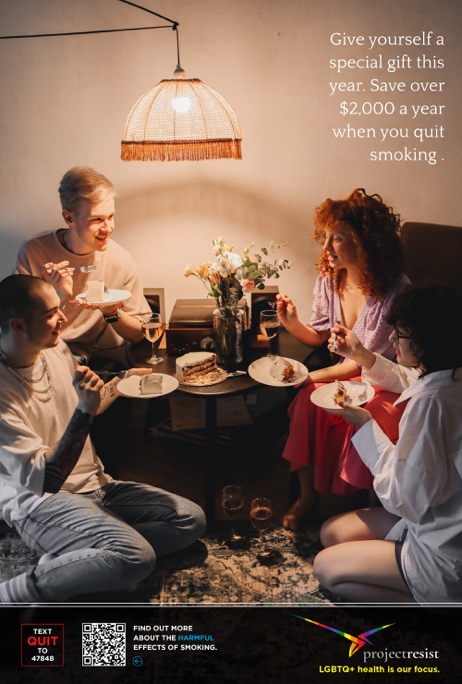* |
| *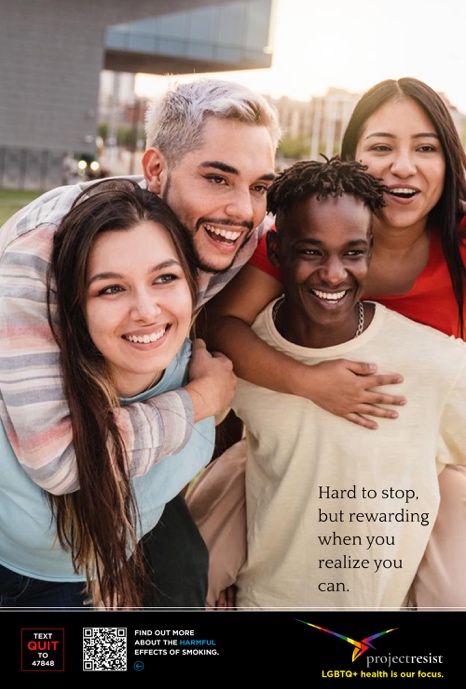* | *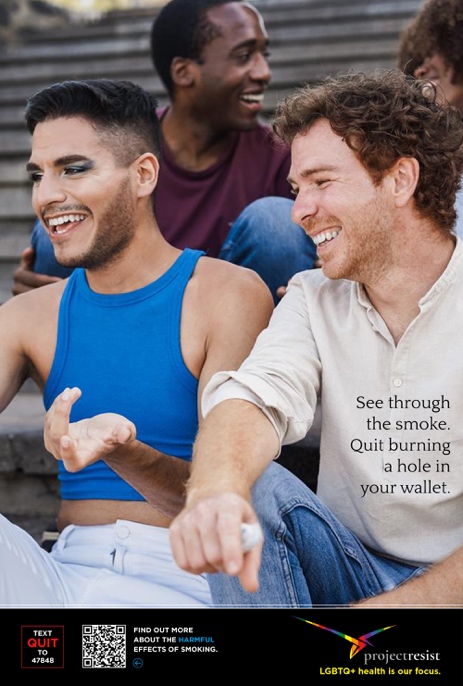* | *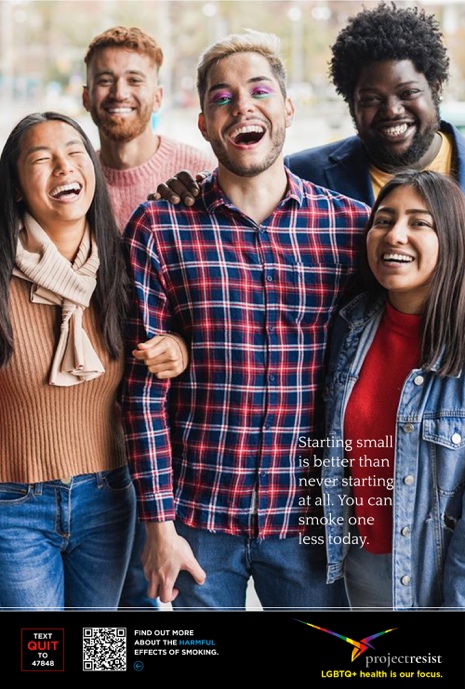* |

*Note.* We selected the six top-ranked images in each gender-expression category from Study 1 based on combined evaluations across four criteria: image liking, character similarity, cultural sensitivity, and minimal anger induction. Anti-smoking arguments were selected based on perceived message effectiveness evaluated in another message pretesting. We selected the six top-ranked arguments from a pool of 40 arguments that received high ratings on perceived message effectiveness and matched each argument to an image.

**Supplementary Materials: Sensitivity Analysis**

**Table S9**

Sensitivity Analysis: Effects of Image Gender Expression and Respondent Gender Identity on Perceived Targetedness (Study 1)

|  | Main Effect | | Interaction Effect | |
| --- | --- | --- | --- | --- |
|  | *B* | *SE* | *B* | *SE* |
| Intercept | 2.69^***^ | 0.34 | 2.93^***^ | 0.35 |
| Gender expression (Referent: Transgender/nonbinary expressions) |  |  |  |  |
| Feminine expressions | −0.01 | 0.08 | −0.16 | 0.10 |
| Masculine expressions | −0.15^+^ | 0.08 | −0.51^***^ | 0.11 |
| Multiple gender expressions | 0.08 | 0.10 | −0.12 | 0.12 |
| Respondent gender identity (Referent: Gender-expansive identity) |  |  |  |  |
| Trans feminine | −0.13 | 0.25 | 0.03 | 0.31 |
| Trans masculine | −0.06 | 0.16 | −0.07 | 0.20 |
| Woman (cisgender SMW) | −0.03 | 0.09 | −0.25^*^ | 0.12 |
| Man (cisgender SMM) | −0.41^***^ | 0.10 | −0.73^***^ | 0.12 |
| LGBT image perception | 0.48^***^ | 0.05 | 0.47^***^ | 0.05 |
| Gay or lesbian image perception | 0.16^***^ | 0.05 | 0.17^***^ | 0.05 |
| Bisexual image perception | 0.22^***^ | 0.05 | 0.21^***^ | 0.05 |
| Number of characters | 0.05^*^ | 0.02 | 0.06^*^ | 0.02 |
| Age | 0.01 | 0.01 | 0.01 | 0.01 |
| Sexual orientation |  |  |  |  |
| Gay or lesbian | 0.33^**^ | 0.10 | 0.33^**^ | 0.10 |
| Other | −0.05 | 0.08 | −0.05 | 0.08 |
| Race |  |  |  |  |
| Black | −0.12 | 0.11 | −0.12 | 0.11 |
| Other | −0.06 | 0.08 | −0.06 | 0.08 |
| Education |  |  |  |  |
| Some college | −0.03 | 0.10 | −0.02 | 0.10 |
| Bachelor’s degree | 0.17 | 0.11 | 0.17 | 0.11 |
| Graduate degree | −0.06 | 0.18 | −0.02 | 0.18 |
| Income | −0.00 | 0.02 | −0.00 | 0.02 |
| Marital status | 0.00 | 0.10 | 0.00 | 0.10 |
| Smoker status |  |  |  |  |
| Occasional smoker | −0.26 | 0.16 | −0.26 | 0.16 |
| Ex-smoker | −0.36^*^ | 0.16 | −0.37^*^ | 0.16 |
| Someone who tried smoking | −0.09 | 0.16 | −0.11 | 0.16 |
| Non-smoker | −0.26^*^ | 0.13 | −0.28^*^ | 0.13 |
| Interaction |  |  |  |  |
| Trans feminine × Feminine expressions | ⎯ | ⎯ | −0.02 | 0.31 |
| Trans feminine × Masculine expressions | ⎯ | ⎯ | −0.45 | 0.30 |
| Trans feminine × Multiple gender expressions | ⎯ | ⎯ | −0.08 | 0.31 |
| Trans masculine × Feminine expressions | ⎯ | ⎯ | −0.26 | 0.21 |
| Trans masculine × Masculine expressions | ⎯ | ⎯ | 0.20 | 0.20 |
| Trans masculine × Multiple gender expressions | ⎯ | ⎯ | 0.06 | 0.20 |
| Woman (cisgender SMW) × Feminine expressions | ⎯ | ⎯ | 0.38^***^ | 0.11 |
| Woman (cisgender SMW) × Masculine expressions | ⎯ | ⎯ | 0.23^+^ | 0.12 |
| Woman (cisgender SMW) × Multiple gender expressions | ⎯ | ⎯ | 0.26^*^ | 0.12 |
| Man (cisgender SMM) × Feminine expressions | ⎯ | ⎯ | 0.13 | 0.11 |
| Man (cisgender SMM) × Masculine expressions | ⎯ | ⎯ | 0.84^***^ | 0.12 |
| Man (cisgender SMM) × Multiple gender expressions | ⎯ | ⎯ | 0.34^**^ | 0.12 |
| Random components: Variance |  |  |  |  |
| Image level | 0.04 |  | 0.04 |  |
| Individual level | 0.43 |  | 0.44 |  |
| Residual | 0.83 |  | 0.81 |  |
| *N* (total observations) | 3685 |  | 3685 |  |
| *N* (image) | 80 |  | 80 |  |
| *N* (individual) | 461 |  | 461 |  |

*Note*. All coefficients are unstandardized. Gender expression: Transgender/nonbinary expressions is reference category. Respondent gender identity: Gender-expansive identity is reference category. LGBT image perception: No is reference category. Gay or lesbian image perception: No is reference category. Bisexual image perception: No is reference category. Sexual orientation: Bisexual is reference category. Race: White is reference category. Education: High school or less is reference category. Marital status: Married or living as married is reference category. Smoker status: Smoker is reference category. Age, income, and number of characters are treated as continuous variables. ^+^ *p* < .08, ^*^ *p* < .05, ^**^ *p* < .01, ^***^ *p* < .001.

**Table S10**

Sensitivity Analysis: Effects of Message Gender Expression and Respondent Gender Identity on Perceived Targetedness (Study 2)

|  | Main Effect | | Interaction Effect | |
| --- | --- | --- | --- | --- |
|  | *B* | *SE* | *B* | *SE* |
| Intercept | 2.15^***^ | 0.25 | 2.32^***^ | 0.26 |
| Gender expression (Referent: Transgender/nonbinary expressions) |  |  |  |  |
| Feminine expressions | 0.04 | 0.07 | −0.14 | 0.14 |
| Masculine expressions | −0.07 | 0.08 | −0.35^*^ | 0.15 |
| Multiple gender expressions | 0.13 | 0.08 | −0.14 | 0.14 |
| Respondent gender identity (Referent: Gender-expansive identity) |  |  |  |  |
| Trans feminine | 0.02 | 0.18 | −0.16 | 0.37 |
| Trans masculine | −0.06 | 0.13 | −0.25 | 0.24 |
| Woman (cisgender SMW) | 0.03 | 0.07 | −0.14 | 0.13 |
| Man (cisgender SMM) | −0.12 | 0.07 | −0.49^***^ | 0.14 |
| LGBT image perception | 0.53^***^ | 0.12 | 0.52^***^ | 0.12 |
| Gay or lesbian image perception | 0.07 | 0.12 | 0.08 | 0.13 |
| Bisexual image perception | 0.43^***^ | 0.10 | 0.42^***^ | 0.10 |
| Age | 0.01 | 0.01 | 0.01 | 0.01 |
| Sexual orientation |  |  |  |  |
| Gay or lesbian | 0.19^*^ | 0.09 | 0.19^*^ | 0.09 |
| Other | 0.06 | 0.06 | 0.05 | 0.06 |
| Race |  |  |  |  |
| Black | 0.18^+^ | 0.09 | 0.20^*^ | 0.09 |
| Other | −0.03 | 0.06 | −0.04 | 0.06 |
| Ethnicity | 0.06 | 0.08 | 0.07 | 0.08 |
| Education |  |  |  |  |
| Some college | 0.13^+^ | 0.07 | 0.12^+^ | 0.07 |
| Bachelor’s degree | 0.05 | 0.08 | 0.03 | 0.08 |
| Graduate degree | 0.09 | 0.12 | 0.08 | 0.12 |
| Income | 0.00 | 0.01 | 0.00 | 0.01 |
| Smoker status | 0.54^***^ | 0.07 | 0.54^***^ | 0.07 |
| Interaction |  |  |  |  |
| Trans feminine ×  Feminine expressions | ⎯ | ⎯ | 0.19 | 0.55 |
| Trans feminine ×  Masculine expressions | ⎯ | ⎯ | 0.28 | 0.51 |
| Trans feminine ×  Multiple gender expressions | ⎯ | ⎯ | 0.25 | 0.51 |
| Trans masculine ×  Feminine expressions | ⎯ | ⎯ | 0.11 | 0.38 |
| Trans masculine ×  Masculine expressions | ⎯ | ⎯ | 0.36 | 0.32 |
| Trans masculine ×  Multiple gender expressions | ⎯ | ⎯ | 0.18 | 0.40 |
| Woman (cisgender SMW) × Feminine expressions | ⎯ | ⎯ | 0.33^+^ | 0.18 |
| Woman (cisgender SMW) ×  Masculine expressions | ⎯ | ⎯ | 0.18 | 0.19 |
| Woman (cisgender SMW) × Multiple gender expressions | ⎯ | ⎯ | 0.15 | 0.19 |
| Man (cisgender SMM) ×  Feminine expressions | ⎯ | ⎯ | 0.22 | 0.18 |
| Man (cisgender SMM) ×  Masculine expressions | ⎯ | ⎯ | 0.60^**^ | 0.19 |
| Man (cisgender SMM) ×  Multiple gender expressions | ⎯ | ⎯ | 0.63^***^ | 0.19 |
| *F*(*df*) | 10.2^***^  (21, 1167) |  | 7.30^***^  (33, 1155) |  |
| *R*^2^ | .16 |  | .17 |  |
| adj. *R*^2^ | .14 |  | .15 |  |

*Note*. All coefficients are unstandardized. Gender expression: Transgender/nonbinary expressions is reference category. Gender identity: Gender-expansive identity is reference category. Sexual orientation: Bisexual is reference category. Race: White is reference category. Ethnicity: Non-Hispanic is reference category. Education: High school or less is reference category. Smoker status: Not current smoker is reference category. LGBT image perception, gay or lesbian image perception, bisexual image perception, age and income are treated as continuous variables. ^+^ *p* < .08, ^*^ *p* < .05, ^**^ *p* < .01, ^***^ *p* < .001.

**Figure S3**

Sensitivity Analysis: Comparisons of Perceived Targetedness among Image Gender Expressions by Gender Identity (Study 1)


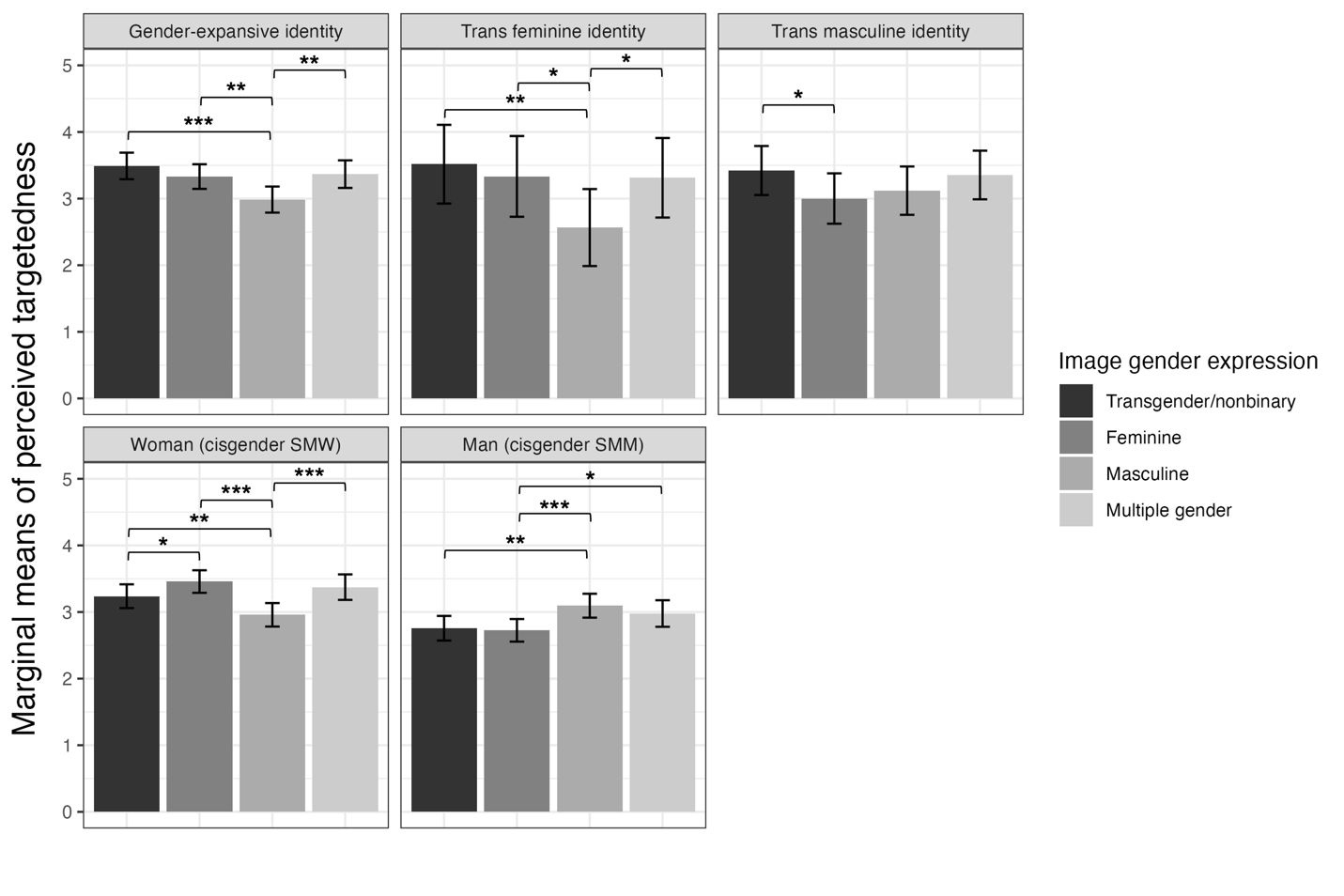


*Note*. ^*^ *p* < .05, ^**^ *p* < .01, ^***^ *p* < .001. The error bars represent 95% confidence interval. The sensitivity analysis additionally included gay or lesbian image perception and bisexual image perception as covariates.

**Figure S4**

Sensitivity Analysis: Comparisons of Perceived Targetedness among Image Gender Expressions by Gender Identity (Study 2)


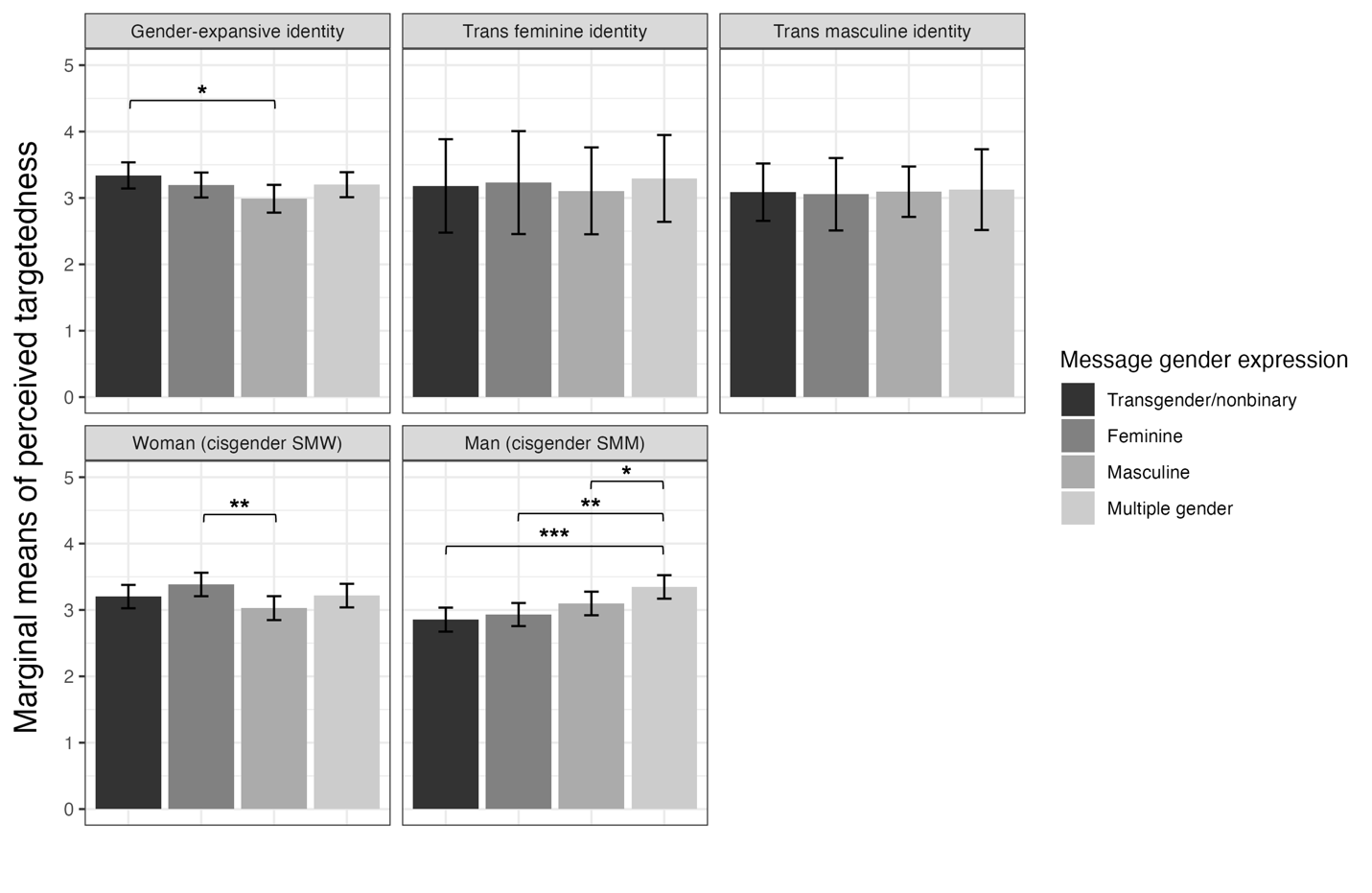


*Note*. ^*^ *p* < .05, ^**^ *p* < .01, ^***^ *p* < .001. The error bars represent 95% confidence interval. The sensitivity analysis additionally included gay or lesbian image perception and bisexual image perception as covariates.
